# Graphene as an Adsorption Template for Studying Double Bond Activation in Catalysis

**DOI:** 10.1021/acs.jpcc.2c02293

**Published:** 2022-08-16

**Authors:** Virginia Boix, Wenbin Xu, Giulio D’Acunto, Johannes Stubbe, Tamires Gallo, Marie Døvre Strømsheim, Suyun Zhu, Mattia Scardamaglia, Andrey Shavorskiy, Karsten Reuter, Mie Andersen, Jan Knudsen

**Affiliations:** † Division of Synchrotron Radiation Research, Department of Physics, Lund University, Sölvegatan 14, 22362 Lund, Sweden; ‡ NanoLund, Lund University, Professorsgatan 1, 22362 Lund, Sweden; § Fritz-Haber-Institut der Max-Planck-Gesellschaft, Faradayweg 4-6, 14195 Berlin, Germany; ∥ Chair for Theoretical Chemistry and Catalysis Research Center, Technische Universität München, Lichtenbergstr. 4, D-85748 Garching, Germany; ⊥ Department of Chemical Engineering, Norwegian University of Science and Technology, Trondheim 7034, Norway; # MAX IV Laboratory, Lund University, Fotongatan 2, 22484 Lund, Sweden; % Aarhus Institute of Advanced Studies, Aarhus University, Aarhus C DK-8000, Denmark; & Department of Physics and Astronomy - Center for Interstellar Catalysis, Aarhus University, Aarhus C DK-8000, Denmark

## Abstract

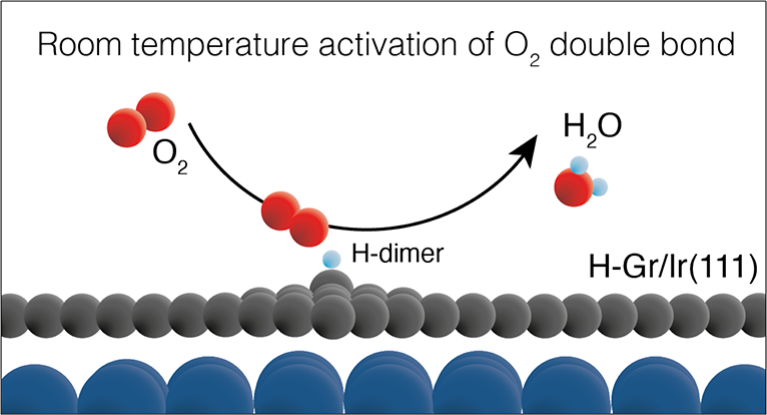

Hydrogenated graphene (H-Gr) is an extensively studied system not only because of its capabilities as a simplified model system for hydrocarbon chemistry but also because hydrogenation is a compelling method for Gr functionalization. However, knowledge of how H-Gr interacts with molecules at higher pressures and ambient conditions is lacking. Here we present experimental and theoretical evidence that room temperature O_2_ exposure at millibar pressures leads to preferential removal of H dimers on H-functionalized graphene, leaving H clusters on the surface. Our density functional theory (DFT) analysis shows that the removal of H dimers is the result of water or hydrogen peroxide formation. For water formation, we show that the two H atoms in the dimer motif attack one end of the physisorbed O_2_ molecule. Moreover, by comparing the reaction pathways in a vacuum with the ones on free-standing graphene and on the graphene/Ir(111) system, we find that the main role of graphene is to arrange the H atoms in geometrical positions, which facilitates the activation of the O=O double bond.

## Introduction

Even though graphene (Gr) is often considered an inert material, intense research efforts have shown that it can be easily modified into a catalyst material.^1−3^ For example, a graphene film can be doped by introducing single atoms or vacancies or by the interaction with the substrate, creating specific catalytic sites. Moreover, one can anchor metal clusters, atoms, or molecules onto graphene and use these anchor sites as active sites for a catalytic reaction.

Anchoring hydrogen atoms to graphene—or hydrogenating graphene—is a simple way to functionalize graphene and potentially form graphene-supported catalytic sites. Hydrogenated graphene (H-Gr) has been extensively studied because of its simplicity as a model system and its relevance for understanding a wide variety of phenomena: from understanding the role of carbonaceous dust grains for catalytic H_2_ formation in astrochemistry to hydrogen storage in carbon-based materials.^4^ Moreover, hydrogenation is also a compelling method to tune the electronic properties of graphene,^5^ to improve the properties of graphene for protective coating of metals,^6^ or to activate graphene for further functionalization.^7,8^

However, the reason for this study of hydrogenated graphene is different from these traditional motivations. Rather than focusing on how H atoms anchored onto graphene modify the catalytic properties of graphene or how a single H atom can potentially act as a catalytic center, we study how the geometrical distribution of H atoms affects the ability to activate the double bond in O_2_ molecules. Or said differently, we use the graphene lattice as a positioning basis to study the ability of different hydrogenation motifs to attack the O=O double bond.

In our study, we use a system consisting of hydrogenated graphene supported on Ir(111) (H-Gr/Ir(111)). The Gr/Ir(111) model system has been extensively studied and is well characterized by many techniques. Its honeycomb structure on hexagonal Ir(111) can be summarized as an incommensurate (9.32 × 9.32) Moiré superstructure with R0° rotation having parallel rows of graphene and Ir(111).^9,10^ The theoretically determined mean height and corrugation of the graphene film is 3.41 and 0.35 Å, respectively, matching experimental values determined from X-ray standing wave experiments very well.^11^ The fingerprint of graphene in X-ray photoelectron spectroscopy (XPS) is a single C 1s peak located at 284.1 eV^12−14^ with the different components caused by the corrugation not usually resolved.^13^

**Figure 1:**
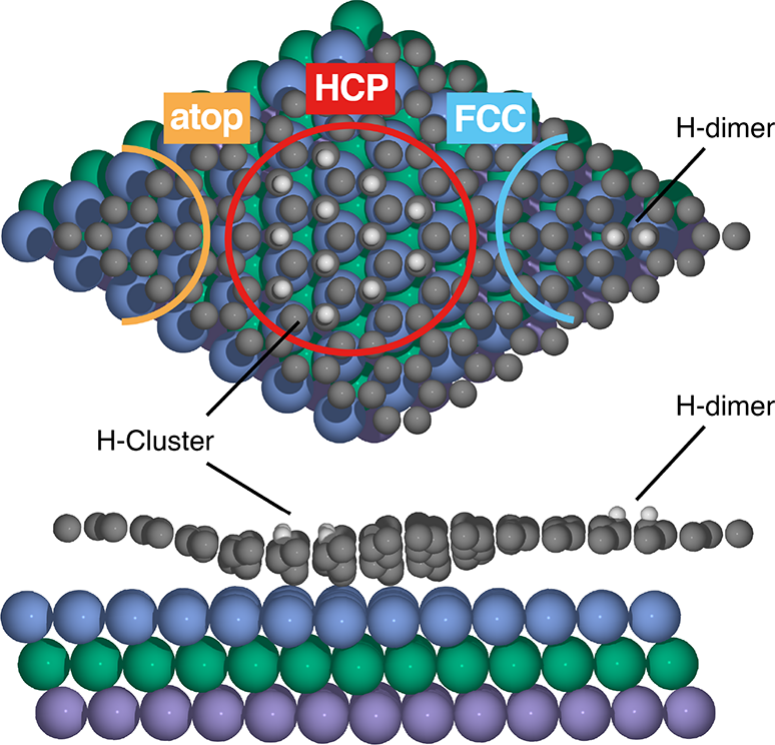
Structure of graphene on Ir(111) functionalized by a 12-atom graphane-like H cluster in the HCP region. The structure of an H dimer in the ortho configuration (here placed in the FCC region) is also sketched. In the top view, the HCP, FCC, and atop regions are highlighted. For ease of visualization, the three Ir layers are shown in different colors. The C atoms are shown in gray, and the H atoms are in white.

Graphene supported by Ir(111) can be hydrogenated both by vibrationally excited H_2_ molecules^6^ or by H radicals,^15,16^ where the latter method is the most common one. As reported by Balog et al.^16^ in 2010, H functionalization of Gr/Ir(111) significantly affects the electronic properties of graphene by opening a bandgap. This publication also demonstrated preferential H radical adsorption in FCC and HCP regions of the Gr/Ir(111) Moiré structure, where the position of every second C atom coincides with an Ir atom below. In this work, we consider such a 12-atom H cluster adsorbed in the HCP region (see Figure 1 for a top and side view of a representative unit cell of the system) since this cluster size has been found to be very stable from DFT calculations.^17^ Similar to metal cluster formation on Gr/Ir(111),^18,19^ the preferred FCC and HCP adsorption regions are explained by a sp^2^ to sp^3^ rehybridization mechanism where every second C atom forms an upward bond to a H atom and every second C atom forms a downward bond to an underlying Ir(111) surface atom. This sp^2^ to sp^3^ rehybridization also results in the pinning of the graphene films under the hydrogen clusters, which in turn reduces the average Gr–Ir(111) distance.^20^ The bonding motif is similar to that found in the fully hydrogenated material graphane,^21^ where the downward bonds to Ir atoms would instead be bonds to H atoms, resulting in hydrogenation at both sides of the graphene sheet. Hence, these clusters are also termed graphane-like clusters.

Later in 2013, Balog et al. revisited the system with a combined XPS, scanning tunneling microscopy (STM), and density functional theory (DFT) study, where they thoroughly studied the uptake of H radicals and H desorption using temperature-programmed XPS.^14^ They showed that two coexisting H-Gr structures are needed for describing H-functionalized graphene: less stable H dimers (removed between 400 and 630 K) and more stable graphane-like clusters formed on the preferred adsorption sites mentioned above (see the original publication for a more extended discussion). Following this finding, Jørgensen et al. exposed Gr/Ir(111) to H radicals at elevated temperatures and demonstrated how this could be used to avoid the formation of H dimers and create highly periodic arrays of graphane-like clusters, resulting in a doped H-Gr/Ir(111) system with reduced band broadening.^17^ In Figure 1, we also sketch the structure of an H dimer in the ortho configuration, which is one of the two stable configurations at graphene/graphite observed in experiments (the other being the para configuration).^22^

To summarize, both the H-Gr/Ir(111) and the Gr/Ir(111) systems are well characterized in terms of STM, XPS, and DFT. In contrast, studies in which H-Gr/Ir(111) is exposed to high pressures of gas molecules are rare in the literature. We are only aware of one study in which H-Gr/Ir(111) was exposed to CO and where it was demonstrated that the H functionalization could be used to protect graphene against CO intercalation by increasing the pressure required for intercalation by at least a factor of 10.^6^ Knowledge of how the different H motifs, such as H dimers and graphane-like clusters, react with probe molecules at higher pressures closer to the pressures used in real heterogeneous catalysis is therefore lacking.

In this work, we use a combination of ambient pressure XPS (APXPS), STM, and DFT to demonstrate that H dimers on Gr/Ir(111) are particularly active for reacting with O_2_ already at room temperature and at millibar pressures. In addition, we will show that the primary role of graphene is to position the H atoms in favorable positions for attacking the O=O double bond. Our study thus opens up for using graphene as a dense positioning matrix to study the catalytic activity of different atomic cluster motifs. The fundamental knowledge in such studies is of direct general relevance for understanding the elementary steps of catalytic reactions in heterogeneous catalysis, including both thermal and electrocatalysis.

## Methods

### Experimental Details

The Ir(111) single crystal was cleaned by cycles of argon sputtering at room temperature (1 kV, 20 min), with subsequent oxygen glow treatment (1 × 10^–7^ mbar, 1000 K, 5 min) and vacuum annealing to above 1100 K. The temperature was measured by a K-type thermocouple attached to the side of the crystal or by a pyrometer. The cleanliness of the surface was verified by either XPS or STM.

The one monolayer (ML) graphene film was grown with a combination of temperature-programmed growth (TPG) (100 s at 10^–6^ mbar of C_2_H_4_ at room temperature followed by flash annealing to 1123 or 1350 K for the XPS or STM experiments, respectively) and chemical vapor deposition (CVD) (25 min at 10^–7^ mbar of C_2_H_4_ with the sample at 1100 K).^23,24^ This growth recipe takes advantage of TPG forming R0° rotated flakes at high temperatures followed by CVD growth at lower temperatures to form a complete graphene layer without any holes. The Gr film completeness was verified either by STM imaging of the surface or by checking carefully for adsorbed CO in XPS, which is known to adsorb if bare Ir(111) patches exist (see Figure S1).

Slightly different graphene qualities were obtained at two different beamtimes, in which hydrogenated graphene films were exposed to O_2_ and H_2_/C_2_H_4_. This is visible by a slightly broader C 1s spectrum of the pristine graphene film in Figure S1b.

**Figure 2:**
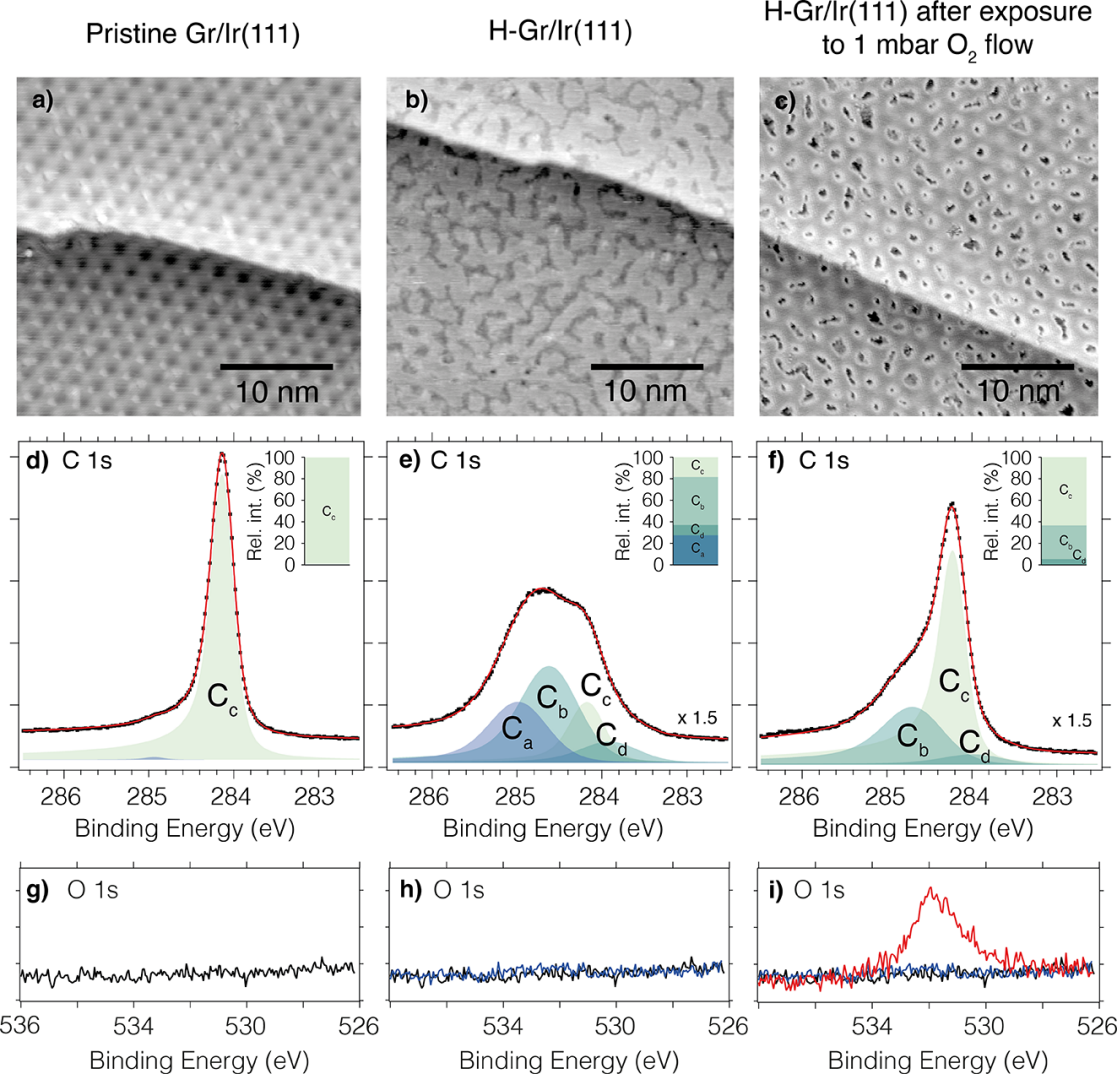
(a–c) 30 × 30 nm^2^ STM images representative of the sample surface with (a) 1 ML Gr/Ir(111) (scanning parameters: *V*_*t*_ = 0.15 V, *I*_*t*_ = 3 nA), (b) Gr/Ir(111) saturated with hydrogen (1 × 10^–7^ mbar of H_2_ for 15 min) (scanning parameters: *V*_*t*_ = −0.95 V, *I*_*t*_ = 1 nA), and (c) H-Gr/Ir(111) after oxygen exposure (1 mbar of O_2_ for 60 s at room temperature) (scanning parameters: *V*_*t*_ = −0.15 V, *I*_*t*_ = 1.5 nA). Additional images can be found in Figure S4. (d–f) C 1s spectrum measured on (d) 1 ML Gr/Ir(111), (e) Gr/Ir(111) saturated with hydrogen (1 × 10^–7^ mbar of H_2_ for 15 min), and (f) H-Gr/Ir(111) after oxygen exposure (1 mbar of O_2_ for 60 s at room temperature). The inset in each panel shows the relative intensities of the C 1s components. See the text for component description. (g–i) O 1s spectra measured on (g) 1 ML Gr/Ir(111), (h) Gr/Ir(111) saturated with hydrogen, and (i) H-Gr/Ir(111) after oxygen exposure.

**Figure 3:**
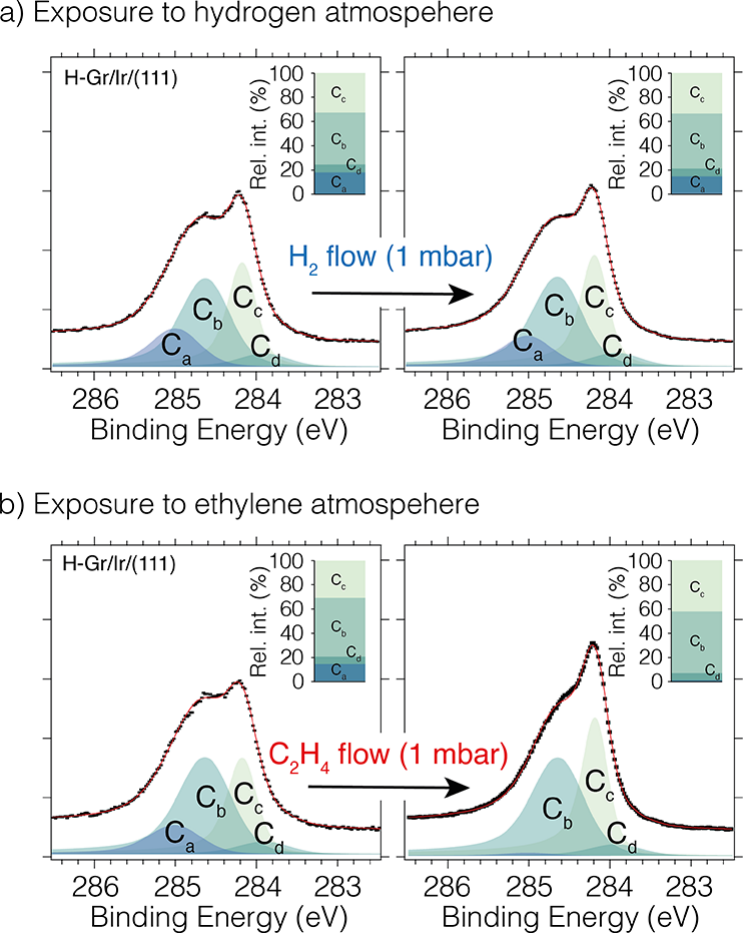
(a) C 1s spectra measured on 1 ML Gr/Ir(111) saturated with hydrogen (5 × 10^–7^ mbar of H_2_ for 15 min), before and after hydrogen exposure (1 mbar of H_2_ for 60 s at room temperature). (b) C 1s spectra measured on 1 ML Gr/Ir(111) saturated with hydrogen (5 × 10^–7^ mbar of H_2_ for 15 min), before and after ethylene exposure (1 mbar of C_2_H_4_ for 60 s at room temperature). The insets show the relative intensity of the fit components for each spectrum.

The 1 ML graphene films were hydrogenated at room temperature by either a HABS MBE-Komponenten hydrogen source (for subsequent millibar O_2_ exposure characterized by STM and XPS) or a Focus EFM hydrogen source (for subsequent millibar H_2_ and C_2_H_4_ exposure characterized by XPS). To reach hydrogen saturated graphene surfaces, the MBE source was operated at 1725–1775 K in a hydrogen partial pressure of 1 × 10^–7^ mbar for 15 min, while the Focus source was operated at 1770 K in a hydrogen partial pressure of 5 × 10^–7^ mbar for 15 min. Note that the O_2_ exposure and the H_2_/C_2_H_4_exposures were performed in separate beamtimes. As different hydrogen sources were used, we obtained slightly different initial hydrogen coverage as the middle panels of Figure S2a demonstrates. Hydrogenation up to saturation was confirmed by comparing the XPS spectra or STM images (Figures 2b,e and 3) with spectra and images from the literature.^14,17^

The hydrogenated graphene was exposed to 1 mbar of O_2_, H_2_, or C_2_H_4_ for 60 s. For the XPS experiments, the exposure was done in the ambient pressure cell (APcell) available at the HIPPIE beamline^25^ at the Max IV Laboratory, Sweden. To avoid beam damage effects, the exposures were performed without the X-ray beam on the sample. For the STM experiments, the oxygen exposure was done in the load lock chamber. In both XPS and STM experiments, the base pressures before the exposure were of the order of 1 × 10^–7^ mbar.

For the XPS experiments, the sample was characterized in vacuum (base pressure 1 × 10^–7^ mbar) by measuring the Ir 4f, C 1s, and O 1s core levels with incident photon energies of 320, 390, and 650 eV, respectively. The same core levels were measured before and after hydrogenation and after the high-pressure exposures to O_2_, H_2_, or C_2_H_4_. All spectra were measured in normal emission. The binding energies were calibrated by recording the Fermi edge immediately after measuring a core level spectrum. Linear background subtraction and the Doniach–Šunjić line shape convoluted with a Gaussian line shape were used in the fitting procedures for the C 1s core levels (Figures 2d–f and 3, Figures S1 and S2). Shirley backgrounds and asymmetric Voight line shapes were used for the analysis of the Ir 4f core levels (Figure S3).

STM images were obtained with a Scienta Omicron STM1 at Lund University. All measurements were performed in constant current mode, at room temperature, and with a base pressure around 1 × 10^–10^ mbar. The scanning parameters used to acquire each STM image are specified in the figure captions (Figures 2a–c and S4).

### Computational Details

All DFT calculations were performed with the Quantum ESPRESSO code (ver. 5.1)^26^ employing the Bayesian error estimation functional with van der Waals correlation (BEEF-vdW)^27^ and scalar-relativistic ultrasoft pseudopotentials taken from the Quantum ESPRESSO pseudopotential database PSLibrary.^28,29^ These pseudopotentials were generated by using the “atomic” code by A. Dal Corso in 2012 (ver. 5.0.2 svn rev. 9415).

The exploration of potential stable intermediates and kinetic barriers in the reaction network was done by using a free-standing (6 × 6) graphene sheet. A (4 × 4) *k*-point sampling was chosen based on a series of convergence tests performed for the hydrogen monomer and dimer adsorption enthalpies.

For further investigations we employed a more realistic system including the Ir substrate. As was also done in a previous DFT study,^17^ we approximated the system using a (8 × 8) graphene lattice over a three-layered (7 × 7) Ir(111) slab, and we adjusted the Ir lattice constant to fit the optimized graphene lattice constant of 2.450 Å. The bottom-most Ir layer was kept fixed in its bulk-truncated positions, and a (1 × 1) *k*-point sampling was employed. The resulting Moiré pattern with its HCP, FCC, and atop regions is shown in Figure 1. Also shown in Figure 1 is the structure of the employed graphane-like cluster. We used a cluster with 12 H atoms situated in the HCP region, as this cluster was found to be very stable in a previous DFT study.^17^

The calculations concerning the H dimer were also performed for adsorption in the HCP region. As we will show later, for the dimer, the interaction with the substrate is very weak, and it is therefore only of minor importance in which region adsorption is considered. Also, a combined X-ray standing wave and DFT study has shown that geometric differences in the adsorption height of graphene over Ir(111) in the different regions are very small.^20^ The graphane-like cluster, however, can only be formed in the HCP or FCC regions due to the requirement that every other C atom must be situated above an Ir atom to allow for bonding to the substrate.

For both the calculations on free-standing and Ir-supported graphene, we employed three-dimensional periodic boundary conditions, and a vacuum region of around 20 Å separated the slab from its periodic images. The cutoff energy was set to 500 and 5000 eV for the orbitals and the charge density, respectively. We employed a Fermi level smearing of 0.1 eV and a dipole correction. All calculations were performed spin-polarized. When needed, for example, for the molecular calculations without the graphene–substrate, we tested several different initial guesses for the spin to check that they converged to the same solution. The structures were relaxed until a maximum force threshold of 0.05 eV/Å was reached.

The transition state energies of reaction steps that were judged important based on the initial exploration of stable intermediates within the water formation reaction network (see structures in Figure S11) were calculated by using the climbing image nudged elastic band (CI-NEB) method.^30^ Moreover, we leveraged the image-dependent pair-potential (IDPP) approach^31^ to create an improved path from initial to final state, instead of simple linear interpolation, where at least seven NEB images were employed for the interpolation.

All energies given are DFT-calculated enthalpies without any thermochemical corrections.

## Results

### Hydrogen Removal from H-Gr

Figure 2 summarizes our STM and XPS characterization of graphene before and after H functionalization and after subsequent exposure to 1 mbar of molecular O_2_ for 60 s at room temperature. Starting with the pristine surface shown in panel a, the STM image shows the characteristic Moiré superstructure of Gr/Ir(111) with a few defects, while the corresponding C 1s spectrum in panel d shows a single C 1s component at 284.11 eV (C_*c*_), in agreement with previous XPS studies of pristine Gr on Ir(111).^12−14^ No other elements than C and Ir were detected in survey spectra (see Figure S1c).

The STM image in Figure 2b was obtained after 15 min of hydrogen functionalization. Inspection of the image shows elongated structures, and the Gr/Ir(111) Moiré superstructure is not visible anymore. Identical structures were observed in previous hydrogenation studies^16^ and were identified as fully saturated H-Gr/Ir(111). Figure 2e shows the corresponding C 1s spectrum measured upon hydrogen functionalization. The spectrum can be fitted with four components already identified in earlier hydrogenation studies:^14,17^ C_*a*_ at 284.99 eV assigned to H dimers, C_*b*_ at 284.64 eV assigned to C atoms binding to H or Ir (due to the graphane-like clusters), C_*d*_ at 283.91 eV assigned to C atoms within and in the near vicinity of the graphane-like clusters, and the already described C_*c*_ component now shifted +50 meV due to the doping of the graphene film. Altogether, we conclude that we obtained a H-saturated graphene, where most of the HCP and FCC regions in the Moiré unit cells are occupied by H clusters, and the rest of the graphene surface is covered by weakly adsorbed H dimers. This conclusion is corroborated by the Ir 4f analysis included in Figure S3.

After characterizing the Gr/Ir(111) surface before and after hydrogenation and demonstrating that the surface contains graphane-like clusters as well as H dimers, we are ready to discuss how chemisorbed hydrogen reacts with different molecules. Panels c and f in Figure 2 show a representative STM image and the corresponding C 1s spectrum acquired after exposure to 1.0 mbar of O_2_ for 60 s at room temperature, respectively.

Starting with the STM image, we observe that the elongated structures clearly visible in Figure 2b are now absent. Instead, single graphane-like clusters are observed in almost every graphene unit cell. Very similar STM images of H-Gr/Ir(111) have been previously reported by Jørgensen et al. after exposing Gr/Ir(111) to atomic hydrogen in a narrow temperature window around 645 K.^17^ In the work of Jørgensen et al. the authors concluded that the clusters preferentially adsorb in the HCP regions of the Moiré superstructure upon atomic hydrogen exposure at high temperatures. Careful inspection of the STM image in Figure 2c shows that the 1.0 mbar of O_2_ exposure also results in clusters adsorbed preferentially in one of the high-symmetry regions, with only a few double clusters in both FCC and HCP regions.

The C 1s spectrum in Figure 2f corroborates the changes observed in the STM image upon O_2_ exposure. Clearly, the C_*a*_ component assigned to H dimers disappears completely, but also the C_*b*_ and C_*d*_ components decrease in intensity from 44% and 10% to 31% and 6%, respectively (see the insets in Figure 2e,f or Figure S2b). At the same time, the C_*c*_ component remains shifted, now at +75 meV higher than the characteristic binding energy of pristine graphene. These observations fit well with the lower H coverage and the single-region occupation of the graphene-like clusters (instead of the combined HCP and FCC occupation before O_2_ exposure) already discussed with the STM images.

It is clear that the number and size of the H clusters are reduced. Whether they are stable at higher O_2_ pressure or not is unknown but would be interesting to probe in future studies. However, a remarkable result of this study is that the dimers are completely absent after 1 mbar of O_2_ exposure. This suggests that the H dimers are particularly prone to be removed by O_2_ exposure at room temperature compared to the graphane-like H clusters. This preference is not surprising because the dimers desorb first at temperatures between 400 and 630 K^14^ and can be fully avoided by hydrogenation at 645 K.^17^ An interesting question to address is, however, whether the two hydrogen atoms in a dimer simply recombine and desorb as H_2_ upon collision with O_2_ molecules or whether hydrogen activates the O=O double bond.

Initial support for the second scenario can be found in the O 1s spectra measured before and after O_2_ exposure (Figure 2h,i). A significant oxygen signal centered at 531 eV appears after oxygen exposure. Etching or intercalation of 1 ML graphene film by molecular oxygen is very unlikely at the pressures and temperatures of the experiment.^32,33^ Moreover, the binding energy of 531 eV does not agree with the reported binding energies for oxygen adsorbed on Ir(111) (530 eV).^33,34^ Instead, the observed binding energy can be identified as epoxy groups on Gr/Ir(111).^35,36^ While we cannot discard the adsorption of oxygen on atomic defects on the graphene film, the presence of an oxygen signal at 531 eV strongly supports the scenario in which oxygen reacts with the H dimers.

In an effort to further investigate the H dimer removal mechanism experimentally, we exposed H-Gr/Ir(111) films to two different molecules: one without any double bonds (H_2_) and one with a double bond (CH_2_=CH_2_). Figure 3 shows the C 1s spectra measured after hydrogenation and after exposure to (a) hydrogen and (b) ethylene. For each spectrum, the relative intensity of the C 1s components is shown as an inset on the top right side (see Figure S2b for the exact values). Upon comparison of panels a and b, it becomes clear that the hydrogen functionalization is essentially unaffected by the H_2_ exposure, while exposure to CH_2_=CH_2_ removes the H dimers. Even though desorption by recombination cannot be fully discarded, these results are in agreement with the proposed scenario where H dimers on H-Gr/Ir(111) can activate unsaturated double bonds while it remains unaffected by H_2_ collisions.

Additional evidence for the reactivity of H dimers can be found in a study by Kyhl et al.^6^ They used the same system (H-Gr/Ir(111)) and exposed it to 1–10 mbar of CO at slightly elevated temperatures (473 K) for intervals of 10–60 min. Upon CO exposure, they observed complete removal of H dimers and a slight decrease of the H clusters components. As in our study, they considered that the H dimers removal is due to a reaction with CO, while the decrease of H clusters coverage is due to the removal of H from the periphery of large graphane-like clusters. Finally, it is worth mentioning that they observed very high stability of the H clusters when the coverage is high enough, even after 60 min exposure at 10 mbar of CO pressure. This is in agreement with H clusters partially withstanding 1 mbar of O_2_ and C_2_H_4_ exposures.

On the basis of the presented experimental evidence and the previous studies found in the literature, we consider that the most plausible scenario for H dimers removal is their reaction with O_2_, CO, and C_2_H_4_. The reaction between H dimers and these molecules should lead to gas-phase reaction products such as, for example, H_2_O in the case of O_2_ exposure. Unfortunately, it is extremely difficult to detect such reaction products experimentally within a 1 mbar atmosphere, as their amount is limited by the coverage of H dimers on the surface. Instead, focusing on the oxygen case, we will use DFT to investigate the possible reaction mechanisms for the activation of the O=O double bond in the following sections.

### DFT Exploration of the Reaction Mechanism

**Figure 4:**
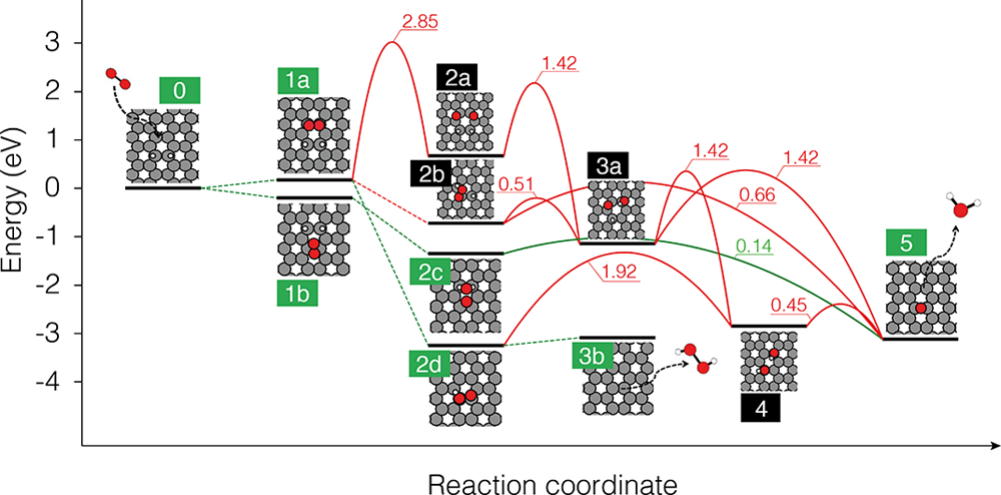
Reaction network for H dimer removal by an O_2_ molecule. DFT-calculated adsorption enthalpies of reaction intermediates (horizontal black lines, referenced to state 0) and kinetic barriers (red or green curves) are shown in eV. Barrierless steps are shown with dashed lines. The most favorable pathways leading to either water (0 → 1b → 2c → 5) or hydrogen peroxide (0 → 1a → 2d → 3b) formation are highlighted in green.

A comprehensive investigation of the reaction mechanism of an H dimer with an O_2_ molecule requires the consideration of all plausible reaction pathways. For this purpose, we performed a systematic sampling of the stability of various reaction intermediates composed of two H atoms and two O atoms on a free-standing graphene sheet. The results are summarized in Figure 4 (larger versions of the structures of all intermediates as well as the transition states can be found in Figures S11–S13).

For the initial state (state 0 in Figure 4), we considered an H dimer in the ortho configuration, which is a stable configuration that has previously been identified on a graphite surface based on STM and DFT,^22^ along with an O_2_ molecule in the gas phase. Physisorption of the O_2_ molecule was found to be most stable in the configuration termed 1b in Figure 4. Both in the gas phase and in state 1b, the O_2_ molecule was found to be in a triplet spin state. For the subsequent reaction steps toward water formation, the following combinations of reaction intermediates were considered, each in various adsorption configurations (see Figures S6–S10 for all calculated structures): (i) two H and two O (2a), (ii) H, O, and OH (3a), (iii) two OH (4), (iv) H and OOH (2b), and (v) HOOH (2d) and (vi) OOH_2_ (2c). These structures are all found to be in the singlet spin state. Note that in the real Gr/Ir(111) system the presence of the metallic substrate would, in any case, quench the spin state of the adsorbates on the Gr surface.

Direct dissociation of the O_2_ molecule results in two H and two O atoms at the graphene surface. The most stable configuration found for these intermediates is the state 2a in Figure 4. From there, subsequent reaction steps could lead to water formation (the final state 5) over state 3a (H, O, and OH) and state 4 (two OH). However, we can exclude this pathway because the calculated barriers are prohibitively large. In particular, the direct O_2_ dissociation step (state 1a → state 2a) has a very high barrier of 2.85 eV, which is incompatible with the experimentally observed room-temperature removal of H dimers.

An alternative mechanism for the activation of the O_2_ bond involves prior reaction with H. In the literature, H-assisted (or associative) pathways have been discussed for many reactions such as CO methanation, Fischer–Tropsch, ammonia synthesis, and electrocatalytic oxygen reduction reaction^37−40^ as a way to overcome the difficulties of activating the strong internal bonds found in, for example, CO, N_2_, or O_2_. Here, we find that the reaction between a physisorbed O_2_ molecule and the H atoms in the dimer is barrierless. The O_2_ molecule can pick up either one H atom (state 2b), two H atoms onto the same O atom (state 2c), or two H atoms onto different O atoms. For state 2d, note that one of the H atoms is not visible in the plot due to its orientation toward the Gr, therefore hidden by the O atom (see Figure S12 for a side view of the structure). From state 2d, the direct desorption of a hydrogen peroxide molecule (H_2_O_2_) is possible with a desorption barrier of 0.16 eV. Dissociation of the OOH molecule formed in state 2b into state 3a has a moderate barrier of 0.51 eV, and the subsequent reaction step to state 4 has an even higher barrier of 1.42 eV. However, much more favorable is the direct water formation from state 2c, for which we calculate a very small barrier of only 0.14 eV.

Referring to Figure 4, direct water desorption from state 2c leaves an O atom on the surface. In a previous study of free-standing graphene by Dai et al.,^41^ the diffusion barrier for an O atom on graphene was calculated to be 0.8 eV. This matches well with our calculated diffusion barriers, which are between 0.64 eV for the largest 8 × 8 supercell tested and 0.82 eV for a smaller 3 × 3 supercell (see Table T1 for details). Taking the value of 0.64 eV as the converged result for an isolated O atom diffusing on a large graphene sheet, we, therefore expect O atoms to be mobile at room temperature. Most likely, the mobile O atoms are stabilized next to the H clusters. Support for this scenario comes from the observation of a small O 1s signal after O_2_ exposure in Figure 2i and from the DFT calculations presented in Figure S14, which show that a single O atom is stabilized next to H atoms.

To sum up, we find that O_2_ can react with the H dimer through H-assisted pathways to form either water (path 0 → 1b → 2c → 5) or hydrogen peroxide (path 0 → 1a → 2d → 3b). Both pathways have low barriers around ∼0.15 eV, which are thus fully compatible with the experimentally observed room temperature reactivity.

### Effects of Cluster Type and Ir Substrate

Having established a low-barrier route for the removal of H dimers on free-standing graphene, we proceed to investigate the role of the Ir substrate as well as the difference between the graphane-like clusters and the H dimers observed in the experiments. Because of the prohibitively large cost of calculating kinetic barriers for the full system with the Ir substrate, we concentrate here on selected reaction intermediates within the most favorable water formation pathway identified in Figure 4 (0 → 1b → 2c → 5).

**Figure 5:**
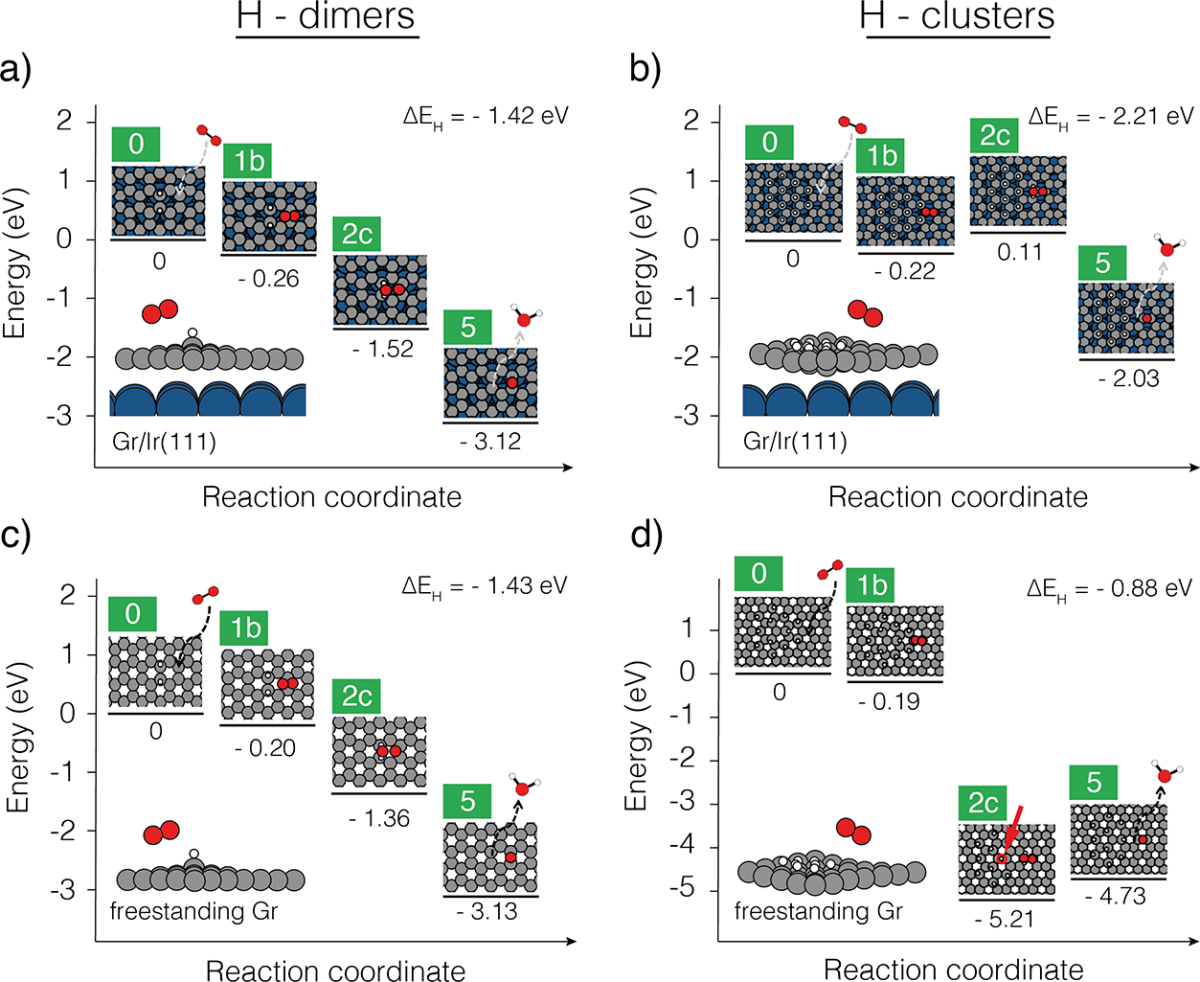
Reaction intermediates from the most favorable water formation pathway highlighted in green in Figure 4 calculated here for (a) the H dimer including Ir substrate, (b) the graphane-like H cluster including Ir substrate, (c) the H dimer without Ir substrate (same as Figure 4), and (d) same H cluster as in (b) but without Ir substrate. The adsorption enthalpy per H atom, Δ*E*_H_, referenced to gas-phase atomic H, is for each cluster given in the upper right corner of the plot.

In Figure 5 we directly compare the stability of these intermediates for H dimers and H clusters with and without the inclusion of the Ir substrate. For the H dimer, we observe that the inclusion of the Ir substrate acts as a very weak perturbation to the reaction intermediates whose adsorption enthalpies are almost unchanged (compare Figures 5a and 5c). We expect only minor perturbations to the kinetic barriers as well because barriers are typically found to be linearly related to the reaction energy according to the Brønsted–Evans–Polanyi principle.^42,43^ Thus, the room temperature removal of H dimers should be possible also for the more realistic full system, in agreement with the experiments.

For the graphane-like cluster investigated in Figure 5b, we let the O_2_ molecule react with two H atoms in the corner of the cluster. We observe that the resulting state 2c is now about 1.6 eV higher in energy compared to the same state for the H dimer in Figure 5a. In fact, this state now has a slightly positive adsorption enthalpy, and the reaction step 1b → 2c can thus no longer be barrierless but must have at least a barrier of 0.33 eV in addition to any kinetic barrier. The reason for this difference is that the H atoms are much more strongly bound in the graphane-like cluster with an average adsorption enthalpy (per H atom) that is about 0.8 eV more stable than in the dimer. This result is in excellent agreement with the experimental observation of preferential removal of the less strongly bound H dimers.

For completeness and to highlight the role of the Ir substrate in the bonding of the graphane-like clusters, we also show the result of the same 12-atom H cluster without the Ir substrate in Figure 5d. Here, state 2c becomes highly favorable. However, this cluster type would never form in reality due to its very low stability with a calculated average H adsorption enthalpy that is about ∼0.55 eV less stable than in the dimer. In fact, the cluster is so unstable that a H atom spontaneously shifted its adsorption site during the geometry optimization of the state 2c (see the highlighted atom in Figure 5d). Our results thus suggest that the deciding factor is the cluster stability, where, in accordance with the Sabatier principle, too low stability excludes the existence of the cluster at the surface and too high stability excludes the formation of the products. On the basis of this, we can also reflect on the type of H dimer investigated. In the calculations presented in this work, we focused on an H dimer in the ortho configuration, but we note that the para configuration is also experimentally observed and has been calculated to have an H adsorption enthalpy that is identical with the ortho configuration.^22^ Given that the cluster stability seems to be the deciding factor, it is likely that room temperature O_2_-induced removal is also possible for H dimers in the para configuration.

Having shown that H dimers can activate an O=O double bond and form water or hydrogen peroxide at room temperature, independently of the presence of the Ir(111) substrate, it is interesting to address the underlying reason for the high activity of the H dimer motif. Close inspection of the preferred reaction path (0 → 1b → 2c → 5 illustrated in Figure 4) shows that when the end of the O_2_ molecule approaches the H dimer and picks up the two hydrogen atoms, the resulting H–O–H angle is equal to 105.3°, which, interestingly, already resembles the geometry of a free water molecule very much (104.5°) (see Figure S13e), while the O–O bond is significantly stretched (1.547 Å) compared to state 1b (1.236 Å), signaling partial bond breaking.

**Figure 6:**
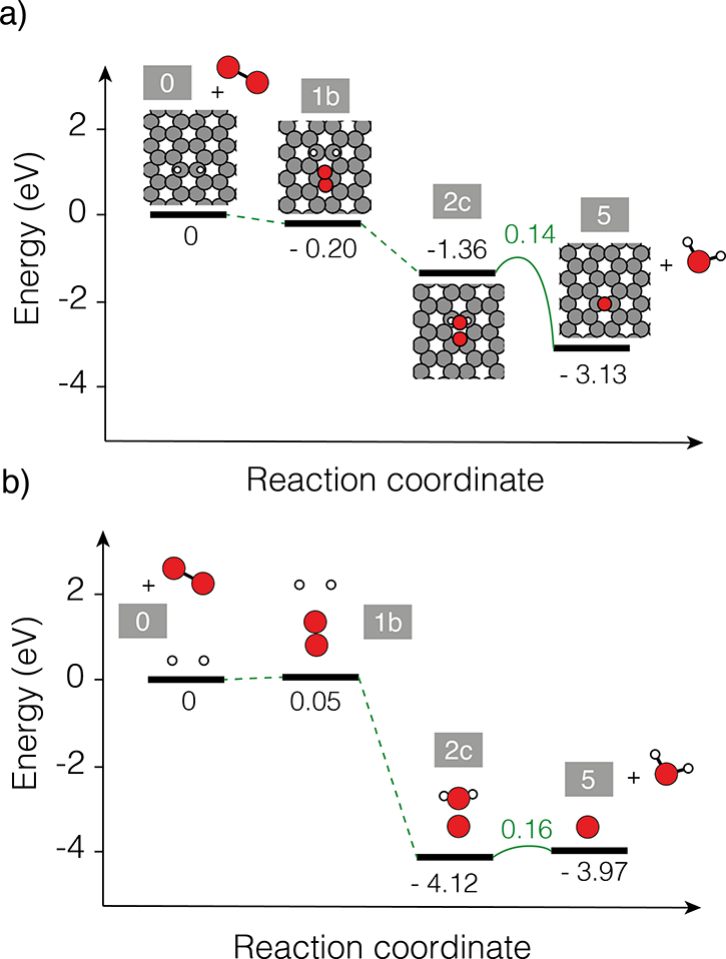
Reaction pathways (a) with graphene (same as Figure 4) and (b) without graphene.

Inspired by this observation and to test if the dimer simply is geometrically optimized for attacking the O=O double bond, we performed an additional computational experiment—impossible to perform experimentally—in which all positions of the O and H atoms are the same as in Figure 4, however, in this case, without any graphene. The recalculated energies of this scenario are shown in Figure 6. Inspection of this figure clearly shows that the geometrical arrangement of the H dimer dictated by the graphene support can attack the O=O double bond, even without the graphene substrate.

## Conclusions

To summarize, we have experimentally proven, both with STM and XPS, that room temperature O_2_ exposure at millibar pressures leads to preferential removal of H dimers on a H-functionalized graphene surface. Using DFT, we showed that the preferential removal of H dimers can be explained by water formation or hydrogen peroxide formation. Focusing on water formation, we find that it is caused by the two H atoms in the dimer motif attacking one end of the physisorbed O_2_ molecule lifting it away from the surface, eventually leading to cleavage of the O=O double bond and water desorption. The reason that the H atoms in clusters are less efficient for attacking physisorbed O_2_ molecules is simply that H atoms in these structures are too strongly bound, making them less active according to the Sabatier principle. Comparing the preferred reaction pathway with identical positions for O and H atoms in a vacuum, on free-standing graphene, and for the graphene/Ir(111) systems reveals qualitatively very similar energetics, suggesting that the main role of graphene is to arrange the H atoms and O_2_ molecule in geometrical positions which facilitates cleavage of the O=O double bond. Therefore, our results suggest that any H-functionalized surface containing a mixture of H clusters and H dimers will consist exclusively of H clusters as soon as the surface has been exposed to millibar pressures of oxygen, which would be the case for air exposure.

Finally, we showed that the ability of the H dimers to activate double bonds is general in nature and not limited to the specific example of the O=O double bond.

## Supporting Information Available

Additional experimental details such as a complete XPS characterization of the samples, additional STM images, and direct comparison of the three experiments included in the article; additional details on DFT such as all calculated adsorption configurations, additional geometrical details on specific dissociation steps, and stability and diffusion barriers for O and H atoms on free-standing graphene (PDF)
