# Supplementary material for: Graphene as an Adsorption Template for Studying Double Bond Activation in Catalysis
Source: J Phys Chem C Nanomater Interfaces. 2022 Aug 16;126(33):14116–24. doi: 10.1021/acs.jpcc.2c02293 (PMC9425632; doi:10.1021/acs.jpcc.2c02293)
Supplement: Supplementary file 1 — jp2c02293_si_001.pdf [file jp2c02293_si_001.pdf]

Supporting Information

Graphene as an adsorption template for studying double bond activation in catalysis

Virginia Boix,[1,2] Wenbin Xu,[3,4] Giulio D'Acunto,[1,2] Johannes Stubbe,[1] Tamires Gallo,[1] Marie Døvre Strømsheim,[5] Suyun Zhu,[6] Mattia Scardamaglia,[6] Andrey Shavorskiy,[6] Karsten Reuter,[3] Mie Andersen,[7,8] Jan Knudsen*[1,2,6]

[1]Division of Synchrotron Radiation Research, Department of Physics, Lund University, Sölvegatan 14, 22362 Lund, Sweden

[2]NanoLund, Lund University, Professorsgatan 1, 22362 Lund, Sweden

[3]Fritz-Haber-Institut der Max-Planck-Gesellschaft, Faradayweg 4-6, 14195 Berlin, Germany

[4]Chair for Theoretical Chemistry and Catalysis Research Center, Technische Universität München, Lichtenbergstr. 4, D-85748 Garching, Germany

[5]Department of Chemical Engineering, Norwegian University of Science and Technology, N- 7491 Trondheim, Norway

[6]MAX IV Laboratory, Lund University, Fotongatan 2, 22484 Lund, Sweden

[7]Aarhus Institute of Advanced Studies, Aarhus University, Aarhus C, DK-8000 Denmark

[8]Department of Physics and Astronomy - Center for Interstellar Catalysis, Aarhus University, Aarhus C, DK-8000 Denmark

S1. Pristine Graphene for O_2 and H_2/C_2H_4 experiments.

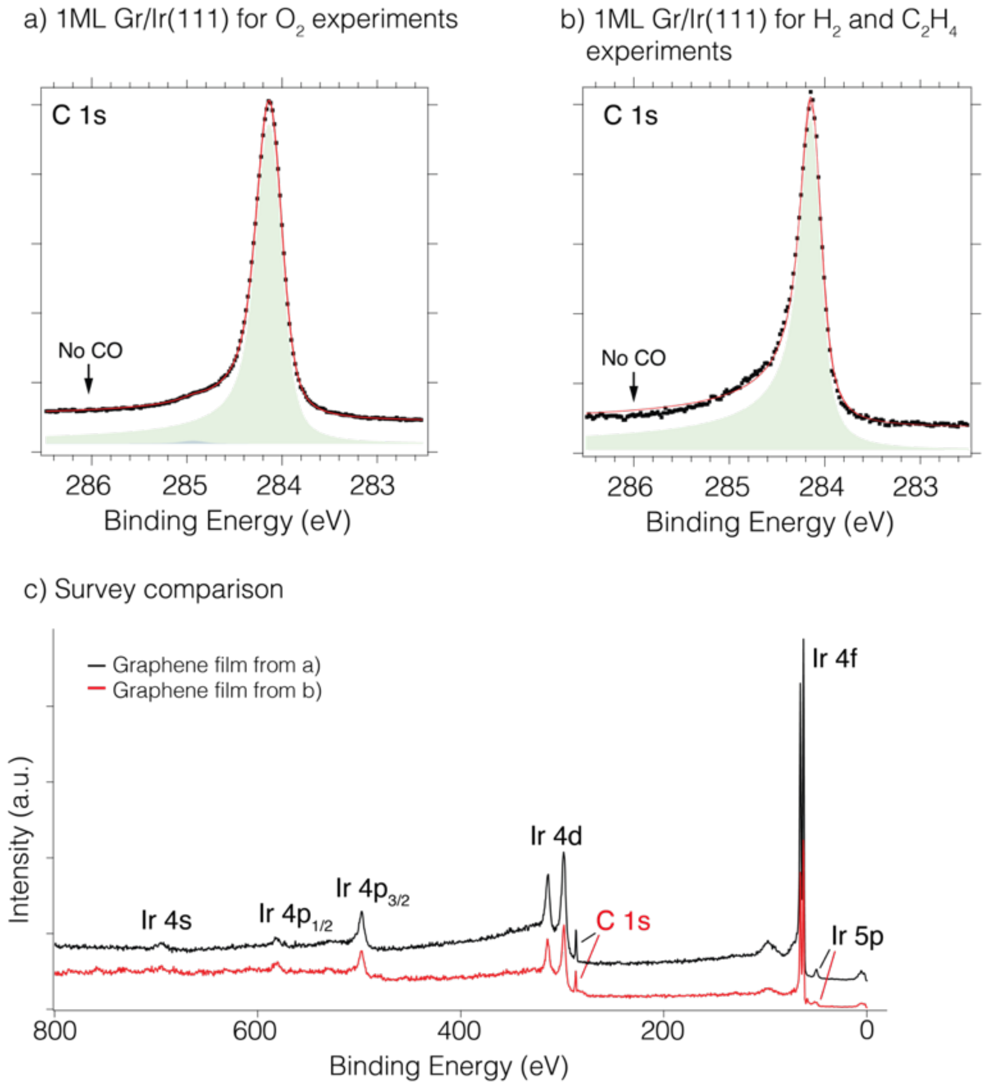

a) C 1s spectrum measured on the graphene film used for the O_2 experiments (figure 2). Spectra measured after exposing the surface to CO, the lack of CO adsorbed signal confirms the film's full coverage. b) C 1s spectrum measured on the graphene film used for the H_2 and C_2H_4 experiments (figure 3), also after CO exposure. The lack of CO adsorbed signal confirms the film's full coverage. c) Survey spectra measured after graphene growth in both cases. Only iridium and carbon can be clearly distinguished. Note that the red spectrum shows oscillations at the high-binding energy side (500 - 800 eV). Those are due to instabilities of the beamline during the measurement.

S2a. Direct comparison: H-Gr exposed to O_2, H_2 and C_2H_4

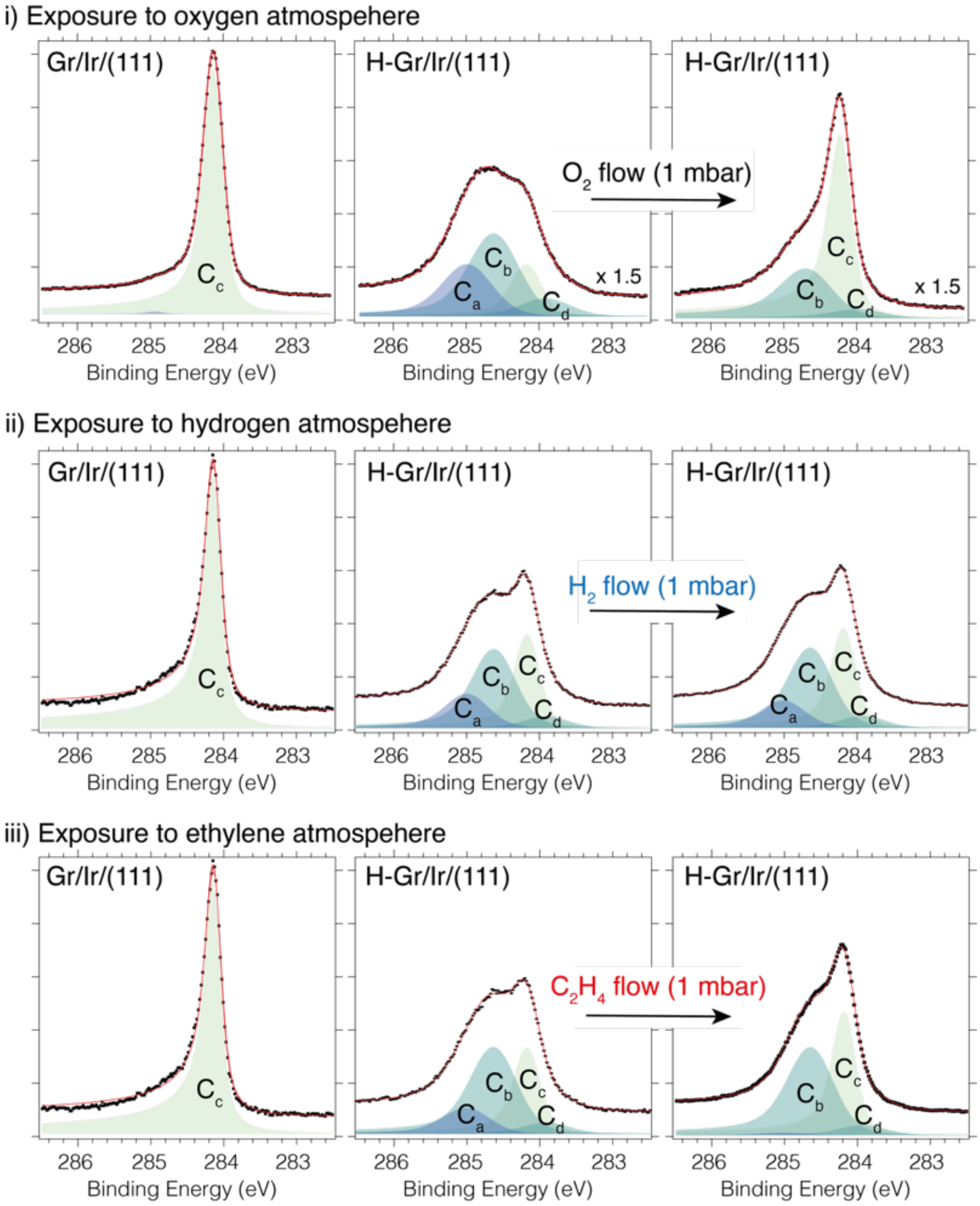

Direct comparison of the C 1s curve fitting after graphene growth, after hydrogenation and after the following exposures: i) 60 s, 1.0 mbar O_2, ii) 60 s, 1.0 mbar H_2, and iii) 60 s, 1.0 mbar C_2H_4. Exposure to O_2 and C_2H_4 results in the complete removal of H dimers (component C_a), while exposure to H_2 leads to no change to the C 1s spectrum. The slightly different relative size of C_a component is caused by the use of different hydrogen sources at different beamtimes (see the experimental section in the main article).

S2b. Direct comparison: H-Gr exposed to O_2, H_2 and C_2H_4

i) Exposure to oxygen atmospehere

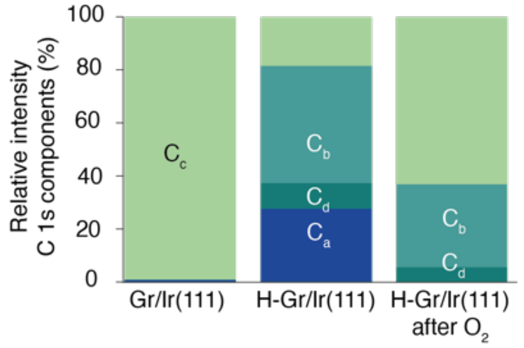

	Pristine Gr	H-Gr	After O_2 exposure
C_c (%)	99	18	63
C_b (%)	0	44	31
C_d (%)	0	10	6
C_a (%)	1	28	0

ii) Exposure to hydrogen atmospehere

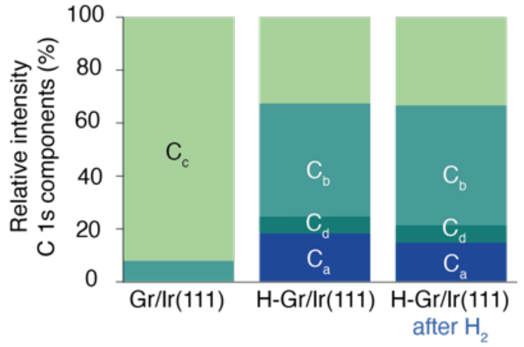

	Pristine Gr	H-Gr	After H_2 exposure
C_c (%)	92	33	35
C_b (%)	7	43	44
C_d (%)	0	6	7
C_a (%)	1	18	15

iii) Exposure to ethylene atmospehere

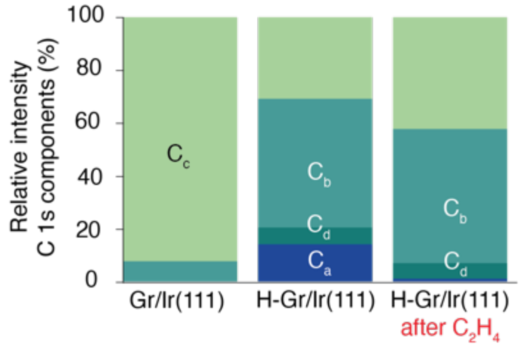

	Pristine Gr	H-Gr	After C_2H_4 exposure
C_c (%)	92	31	42
C_b (%)	7	48	51
C_d (%)	0	6	6
C_a (%)	1	14	1

Accumulative plots representing the intensity of each C 1s component after graphene growth, after hydrogenation, and after i) 60 s O_2 exposure at 1.0 mbar, ii) 60 s H_2 exposure at 1.0 mbar, and iii) 60 s C_2H_4 exposure at 1.0 mbar. The values are stated next to each plot. All fits are shown in figure S2a.

S3. Discussion of H clusters and interaction with the substrate with the analysis of Ir $4f_{7/2}$

In the main text we have shown how the exposure to molecular oxygen of a H saturated Gr film results in the selective removal of the most weakly adsorbed H structures (the H dimers). We observed, however, that some of the more strongly adsorbed H atoms (which form H clusters in the FCC and HCP regions of the moiré) are also removed. While the removal of dimers is not reflected in the Ir$4f_{7/2}$ spectra, the partial removal of H clusters is reflected by the relative intensities of the iridium components.

The Ir $4f_{7/2}$ spectra in figure S3 are fitted with three components: bulk iridium (I_{bulk} at 60.8 eV), surface iridium (I_{surf} at 60.3 eV), and surface iridium atoms binding to C atoms from the graphene film atop (I_c at 60.7 eV). For the fit, we have assumed that the total surface area (I_{surf} + I_c) is maintained, and only the relative intensities of I_{surf} and I_c change.

After the graphene growth, in panel a, we only observe the bulk and surface components, due to the weak interaction between the Ir surface and the graphene film. After hydrogenation, the presence of hydrogen clusters in both FCC and HCP regions of the moiré unit cell increases the interaction of graphene with the substrate. This is reflected in the Ir $4f_{7/2}$ spectra (left spectrum in panels b, c and d), where now approximately 80 % of the I_{surf} intensity becomes I_c, meaning that 80 % of the Ir surface is now binding to C-atoms. This coverage is in agreement with the H-saturated Gr surface described in the main text, where most of the HCP and FCC regions in the moiré unit cells are occupied by H clusters, and the rest of the Gr surface is covered by weakly adsorbed H dimers (figure 2b in the main text). These changes in the Ir $4f_{7/2}$ spectra are also in good agreement with previous studies[1].

After oxygen exposure (right spectrum in figure S3b), the I_{surf} component recovers to 55 %, meaning that only 45 % of the iridium surface atoms remain binding to C-atoms from the graphene atop. This is in agreement with the reduction of the cluster-related C 1s components (C_b and C_d), which decrease from 55% to 36% of the total C 1s intensity. Note that for every C-H bond breaking from the graphene-like clusters, 1 to 3 Ir surface atoms unbind to C atoms (assuming that H atoms at the edges of the H clusters are the ones being removed).

Similar observations can be done for the Ir $4f_{7/2}$ spectra measured before and after exposure to other gases (figure S3c for molecular hydrogen and figure S3d for ethylene). After hydrogen exposure, where no change can be observed in the H coverage (see figure 3a, or S2a and S2b above), the I_c and I_{surf}

components maintain almost the same intensity ratios after exposure. After ethylene, on the other hand, which is capable of removing the H dimers but much less efficient than oxygen at affecting the H clusters coverage (see figure 3b or S2a, panel (iii)), we observe a slight recover of the I_{surf} component (from 20 % to 35 %), which can be directly linked to the 5 % diminishing of the C_b and C_d components in the C 1s spectra shown in figure S2b, panel (iii).

Overall, the analysis of the Ir $4f_{7/2}$ confirms the conclusions drawn in the main text: Exposure to oxygen results in the complete removal of H-dimers and reduction of graphane-like H-clusters, while exposure to molecular hydrogen does not affect the system, and exposure to ethylene results in the removal of H-dimers alone.

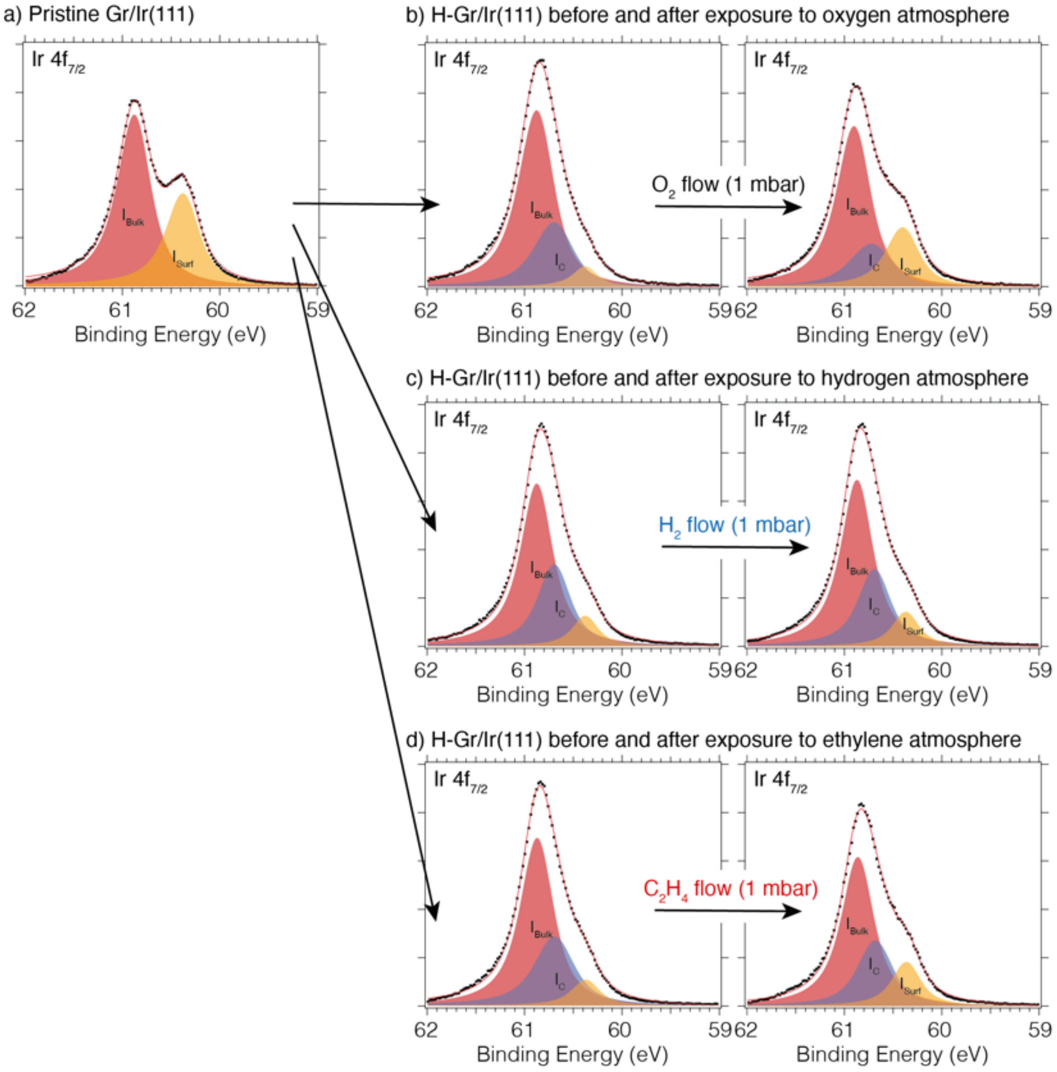

S4. Additional STM images for the O_2 exposure experiment (figure 2)

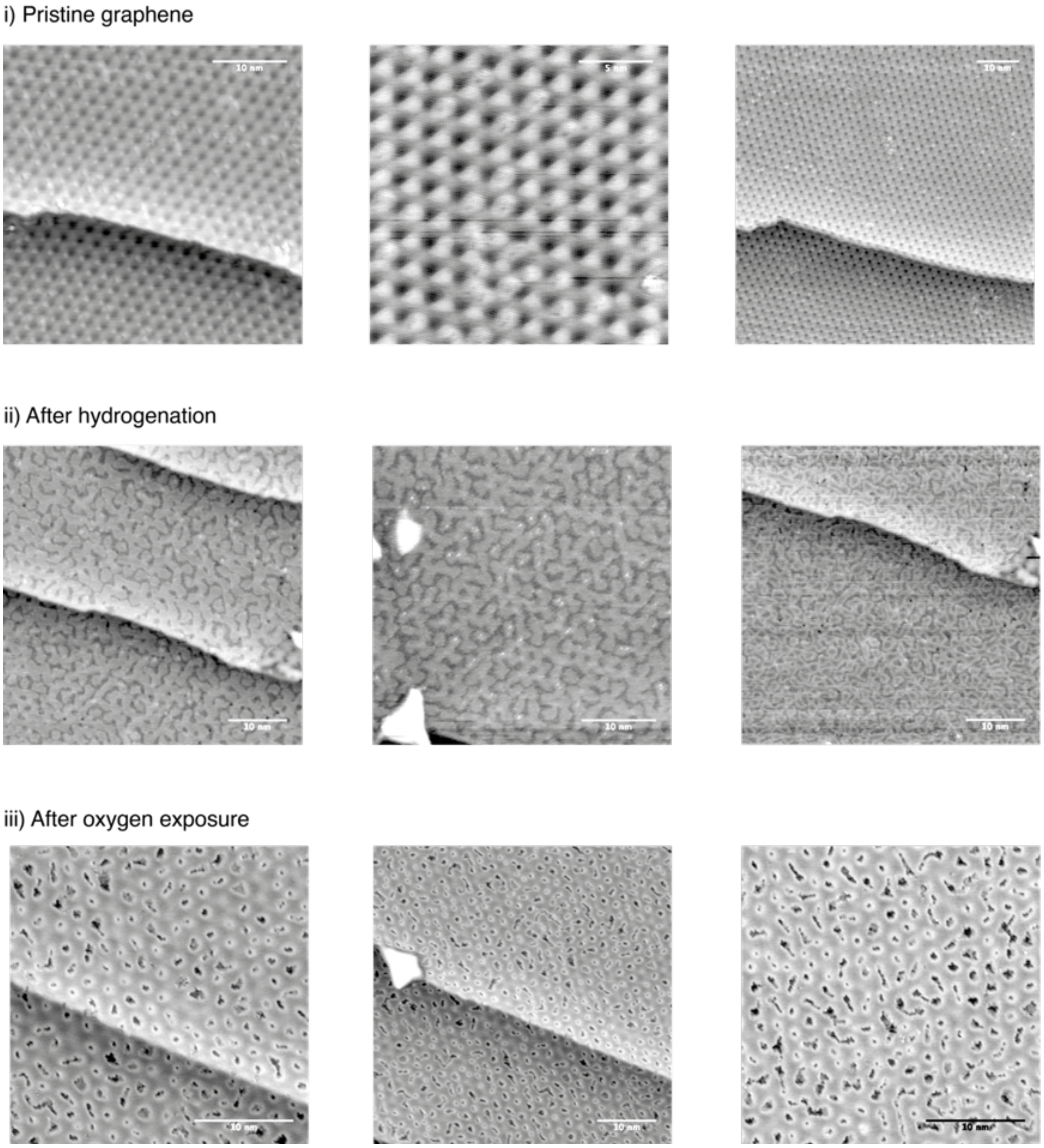

Additional STM images measured on i) pristine graphene, ii) after hydrogenation (H-Gr/Ir(111)), and after exposure to oxygen flow (1.0 mbar, 60 s). Size and scanning parameters: i) (left to right) 40×40 nm^2, V_t = 0.15 V, I_t = 3 nA, 20×20 nm^2, V_t = 0.60 V, I_t = 2 nA, 70×70 nm^2, V_t = 0.15 V, I_t = 3 nA. ii) (left to right) 50×50 nm^2, V_t = - 0.95 V, I_t = 1 nA, 40×40 nm^2, V_t = 1.00 V, I_t = 2.5 nA, 50×50 nm^2, V_t = - 0.95 V, I_t = 1 nA. iii) (left to right) 30×30 nm^2, V_t = - 0.15 V, I_t = 1.5 nA, 50×50 nm^2, V_t = - 0.15 V, I_t = 1.5 nA, 70×70 nm^2, V_t = - 0.15 V, I_t = 1.5 nA. The bright protrusions in the center images of i and ii correspond to impurities on the sample.

S5. Graphene supercell and adsorption sites used for the DFT calculations

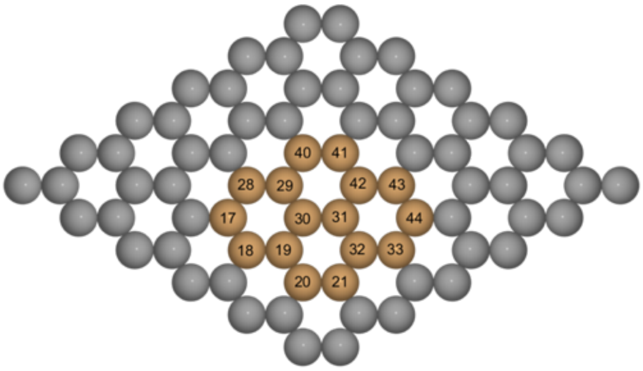

The picture above shows a 6×6 graphene sheet with adsorption sites highlighted in brown color. The annotations are the indices of C atoms onto which adsorption is considered (the others are not used in the calculations). In the following figures S6 - S10, the different investigated adsorption configurations are illustrated with the indices of the adsorption sites and corresponding adsorption strength sorted after the most stable configuration found. The title of each configuration points out where the adsorbates are located in the initial guess structure. Upon DFT relaxation, we filter out all failed structures, e.g. structures which relax into another configuration that is already covered in our systematic testing of adsorption configurations.

S6. Configurations of co-adsorbed 2H and 2O (Para H dimer)

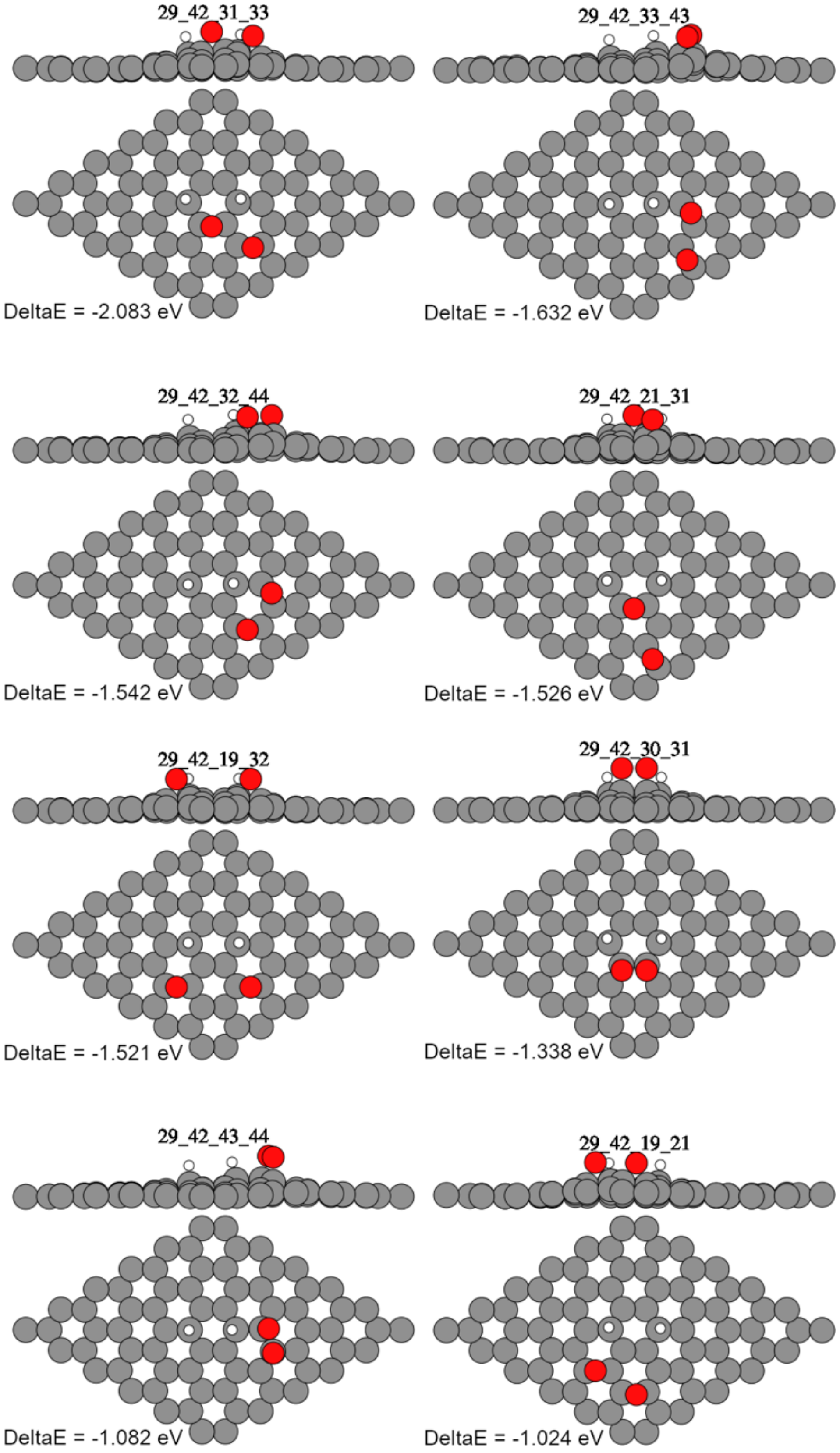

29_42_32_33

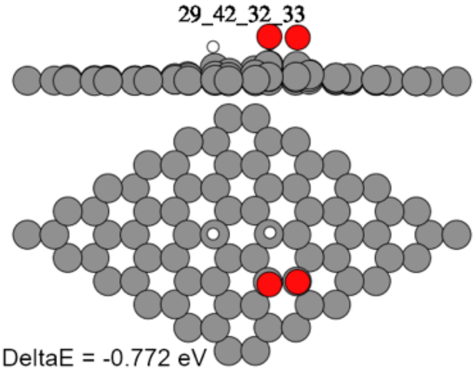

DeltaE = -0.772 eV

29_42_21_32

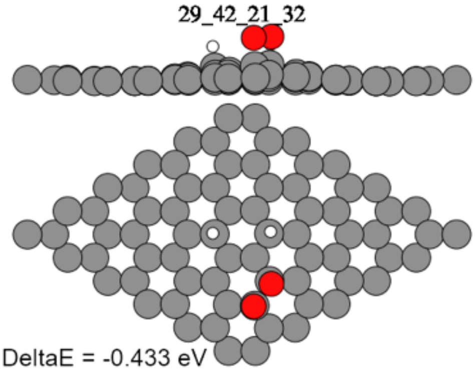

DeltaE = -0.433 eV

29_42_31_32

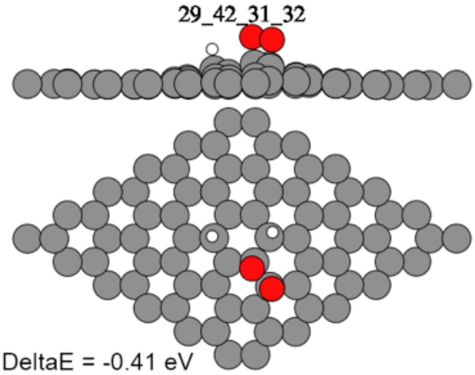

DeltaE = -0.41 eV

29_42_20_21

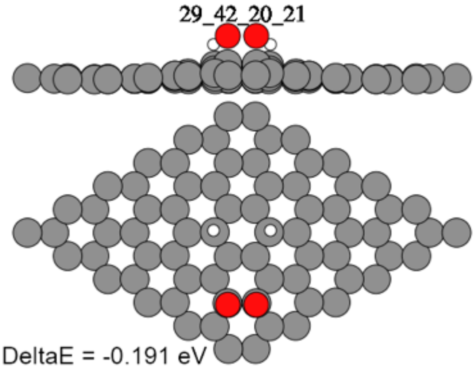

DeltaE = -0.191 eV

29_42_33_44

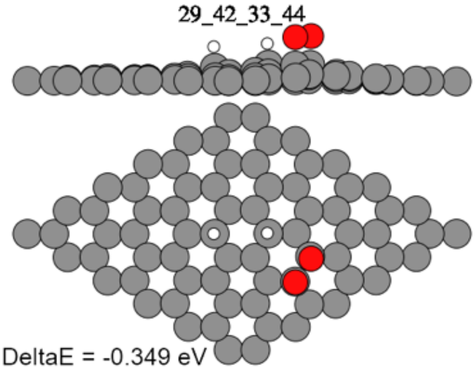

DeltaE = -0.349 eV

S7. Configurations of co-adsorbed 2H and 2O (Ortho H dimer)

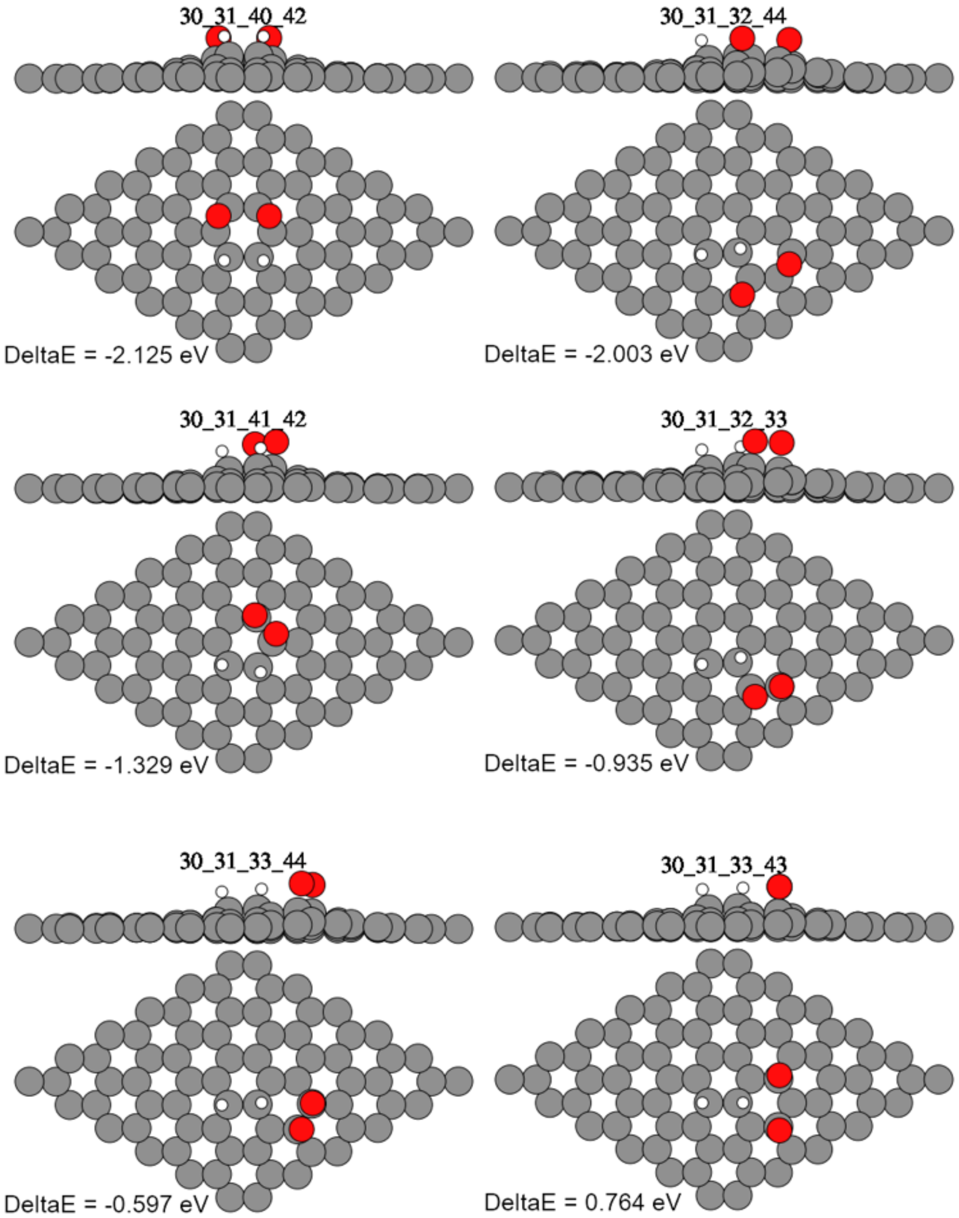

S8. Configurations of co-adsorbed H, O, and OH

20_19_31

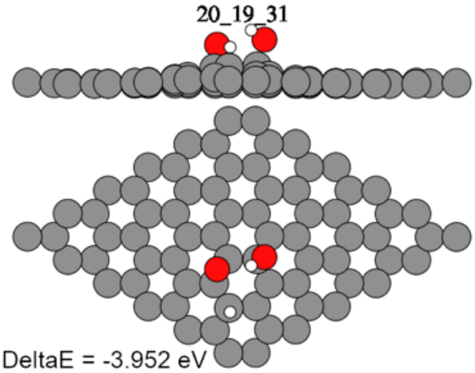

DeltaE = -3.952 eV

20_19_21

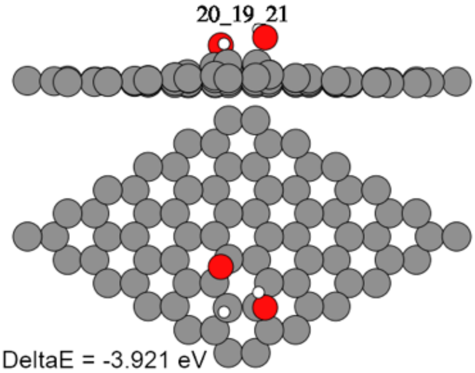

DeltaE = -3.921 eV

21_19_20

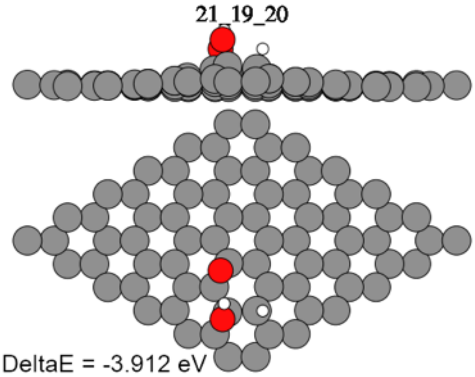

DeltaE = -3.912 eV

19_31_20

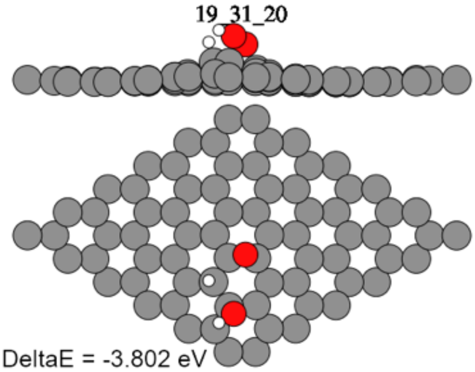

DeltaE = -3.802 eV

19_21_20

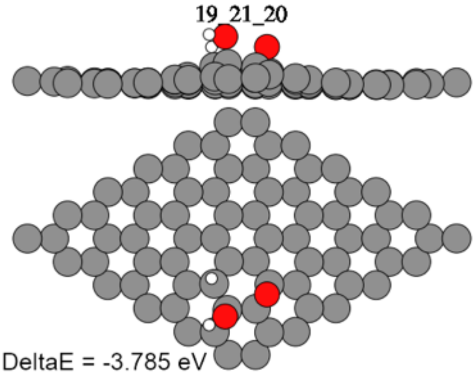

DeltaE = -3.785 eV

20_31_19

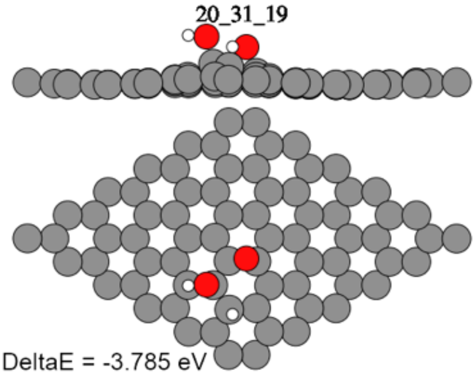

DeltaE = -3.785 eV

30_19_41

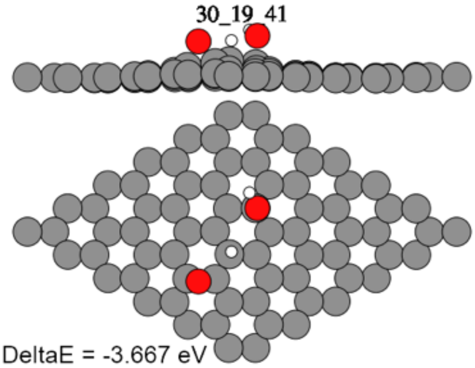

DeltaE = -3.667 eV

41_19_30

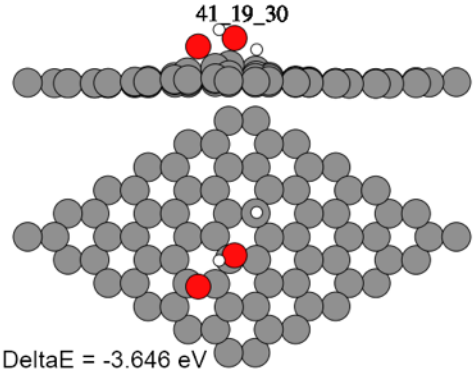

DeltaE = -3.646 eV

20_19_41

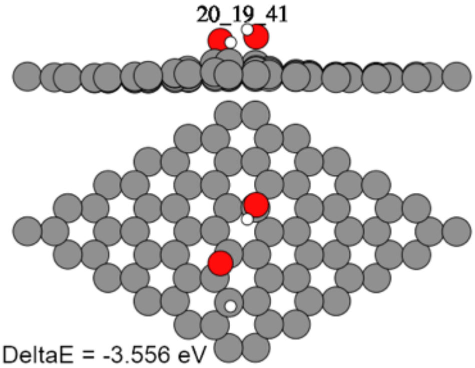

DeltaE = -3.556 eV

41_19_20

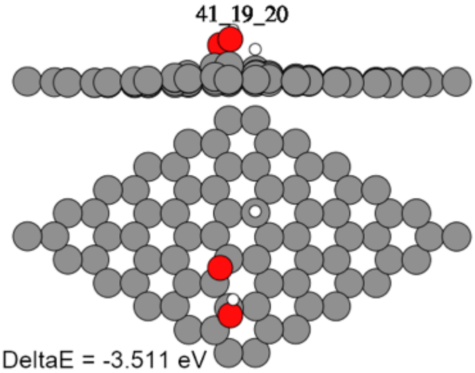

DeltaE = -3.511 eV

30_41_19

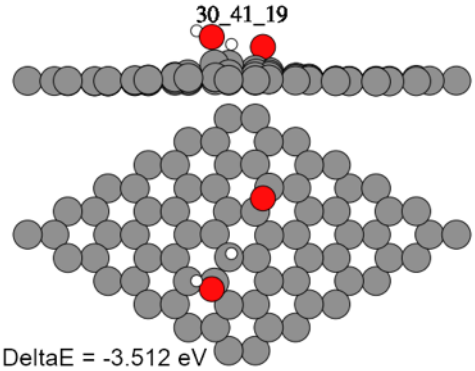

DeltaE = -3.512 eV

19_41_30

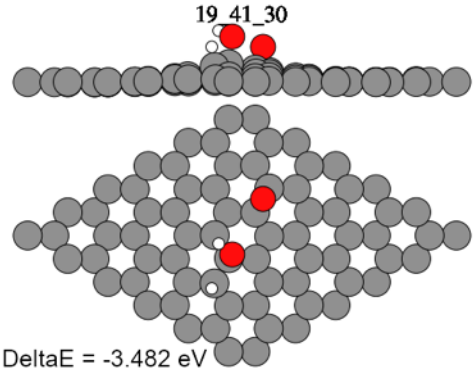

DeltaE = -3.482 eV

32_29_19

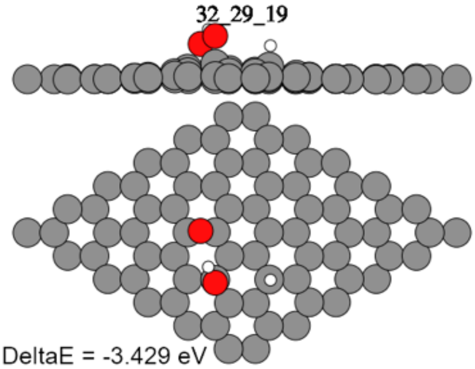

DeltaE = -3.429 eV

19_29_32

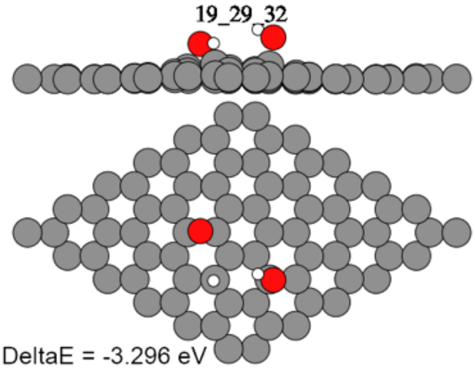

DeltaE = -3.296 eV

20_41_19

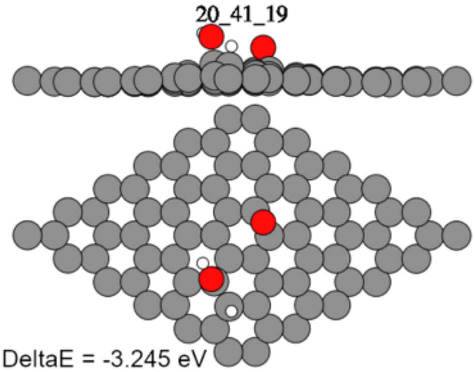

DeltaE = -3.245 eV

32_19_29

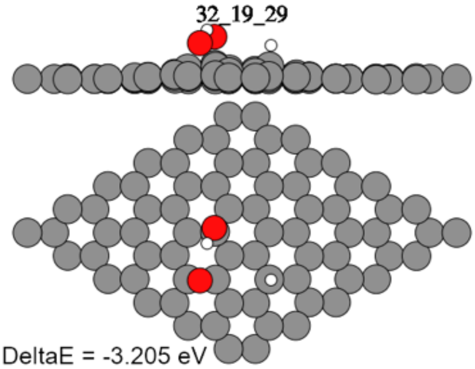

DeltaE = -3.205 eV

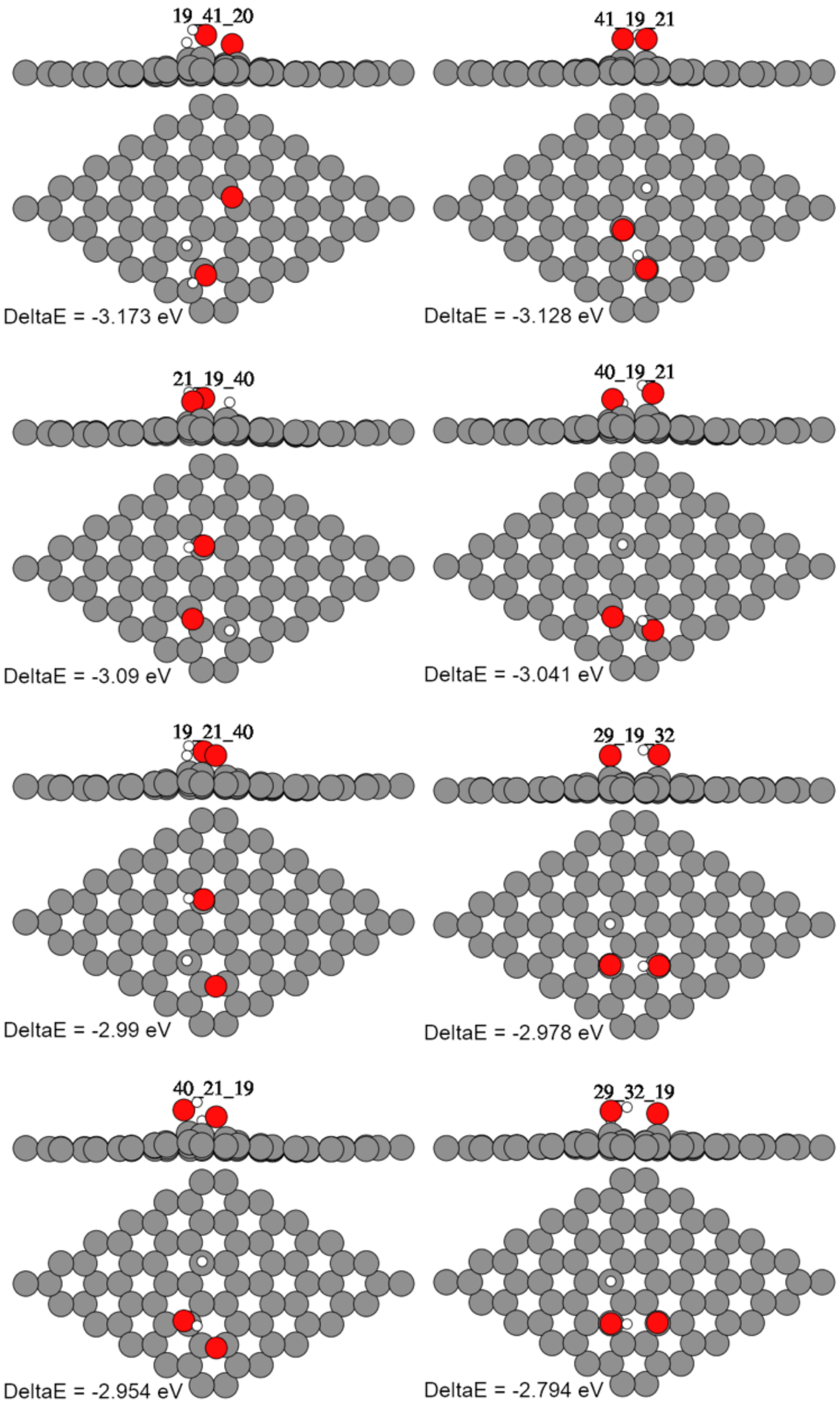

19_41_20
DeltaE = -3.173 eV
41_19_21
DeltaE = -3.128 eV
21_19_40
DeltaE = -3.09 eV
40_19_21
DeltaE = -3.041 eV
19_21_40
DeltaE = -2.99 eV
29_19_32
DeltaE = -2.978 eV
40_21_19
DeltaE = -2.954 eV
29_32_19
DeltaE = -2.794 eV

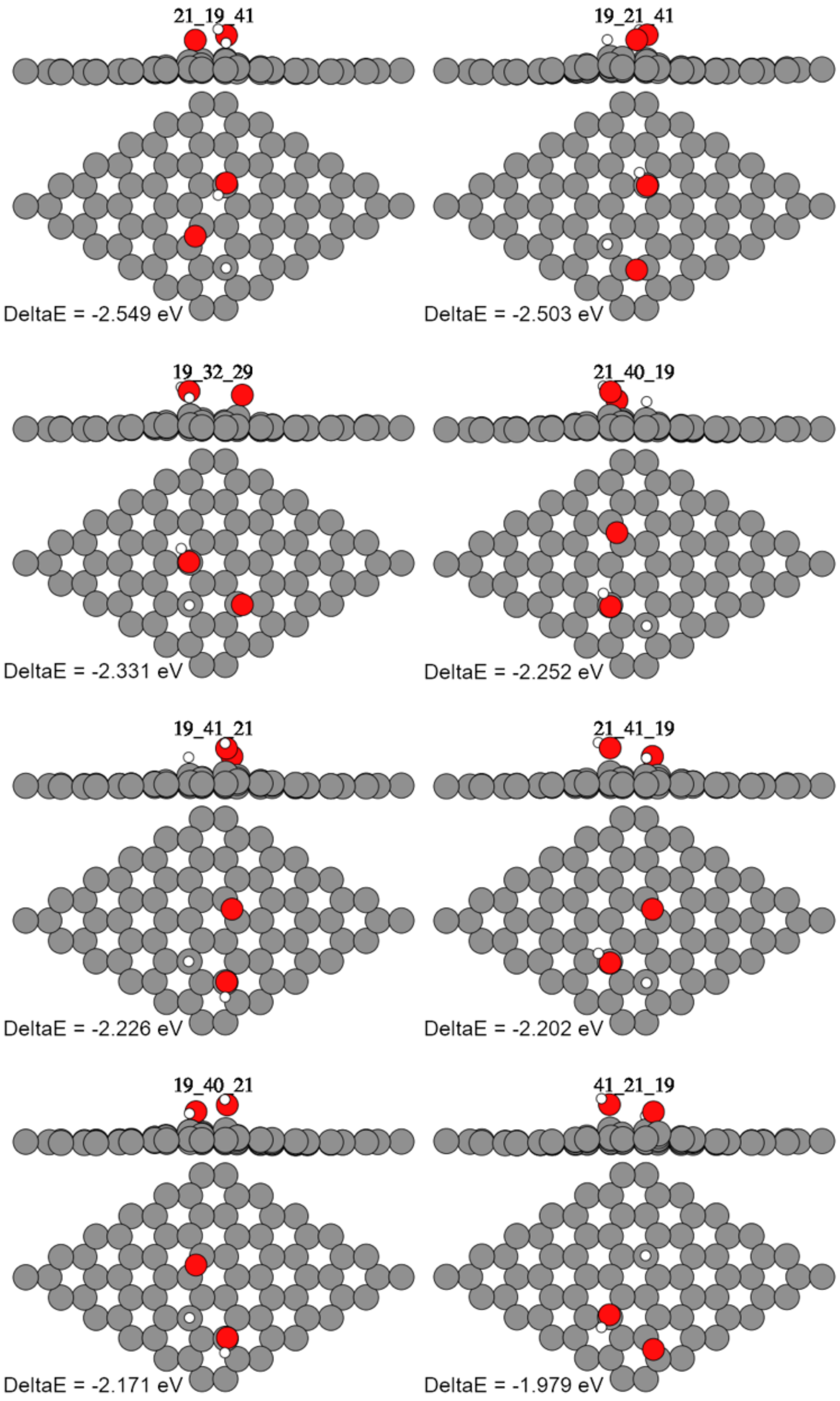

21_19_41
DeltaE = -2.549 eV
19_21_41
DeltaE = -2.503 eV
19_32_29
DeltaE = -2.331 eV
21_40_19
DeltaE = -2.252 eV
19_41_21
DeltaE = -2.226 eV
21_41_19
DeltaE = -2.202 eV
19_40_21
DeltaE = -2.171 eV
41_21_19
DeltaE = -1.979 eV

S9. Configurations of co-adsorbed OH and OH

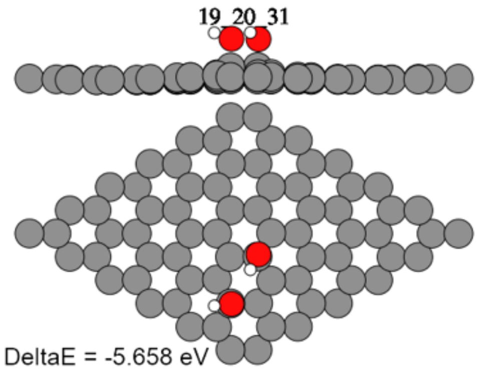

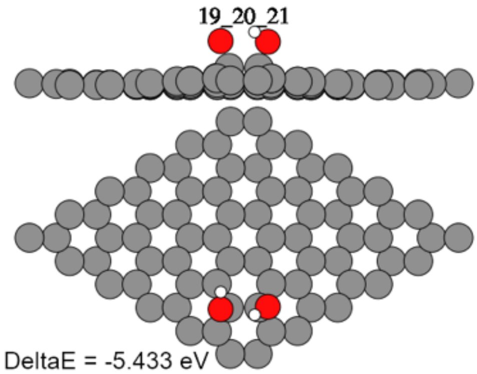

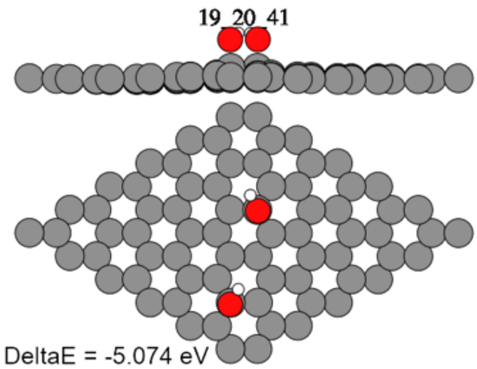

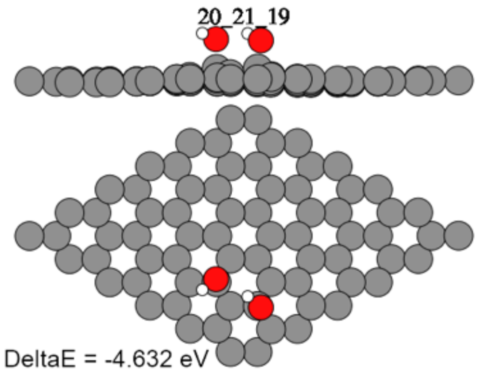

S10. Configurations of co-adsorbed OOH

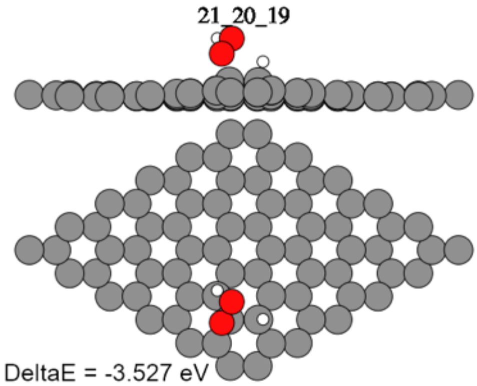

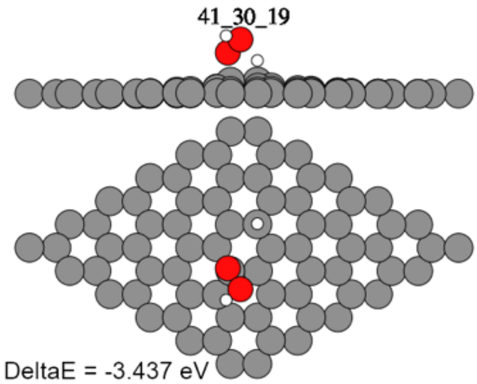

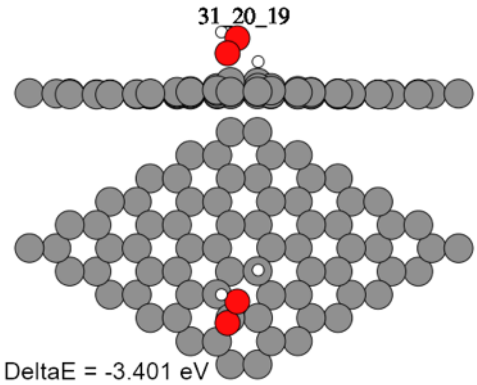

S11. Structures of transition states identified for the reaction network in Figure 3

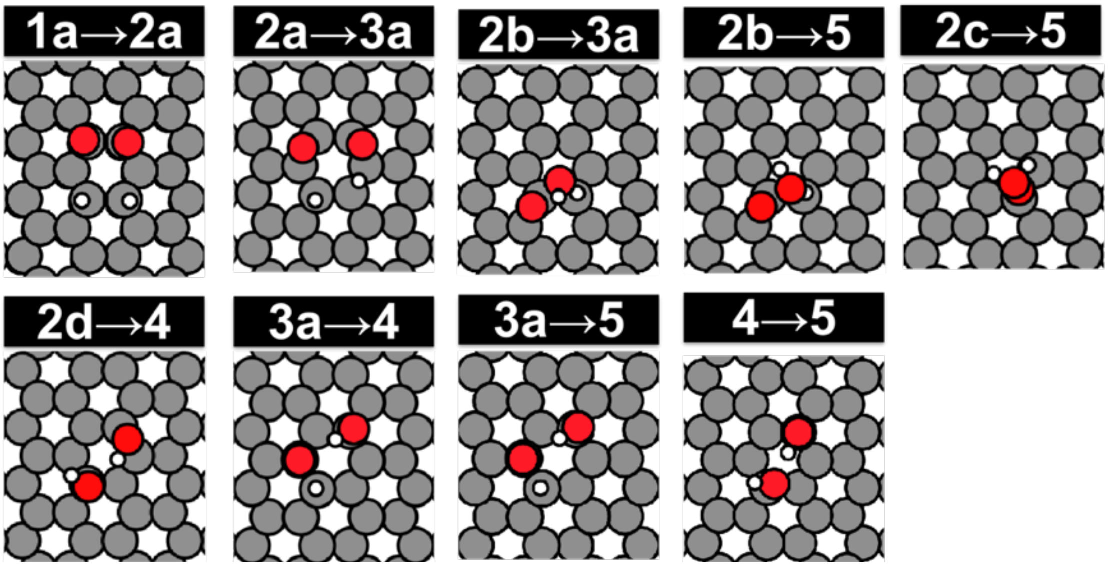

S12. Additional geometric details of state 2d (top and side views)

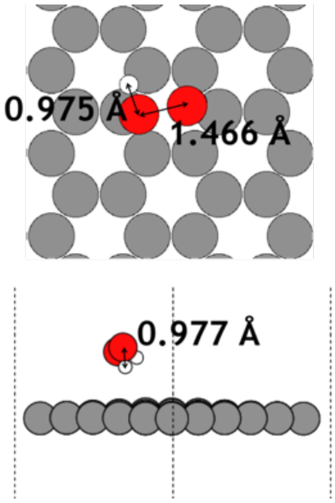

S13. Reaction path 1b to 2c

The structures (top and side views) along the reaction path of 1b => 2c are shown below with some concrete geometry information labeled. Starting from a physisorbed O_2 molecule together with H dimer on the graphene, we observe that O_2 approaches the H dimer and picks up two hydrogen atoms sequentially during the reaction. Specifically, the O=O bond is weakened, as evidenced by the O-O bond length increase to 1.38 and 1.55 Å, respectively, for the first and second hydrogenation steps. Finally, we end up with a H-O-H angle equal to 105.3°, which is very similar to its counterpart in a water molecule (104.5°).

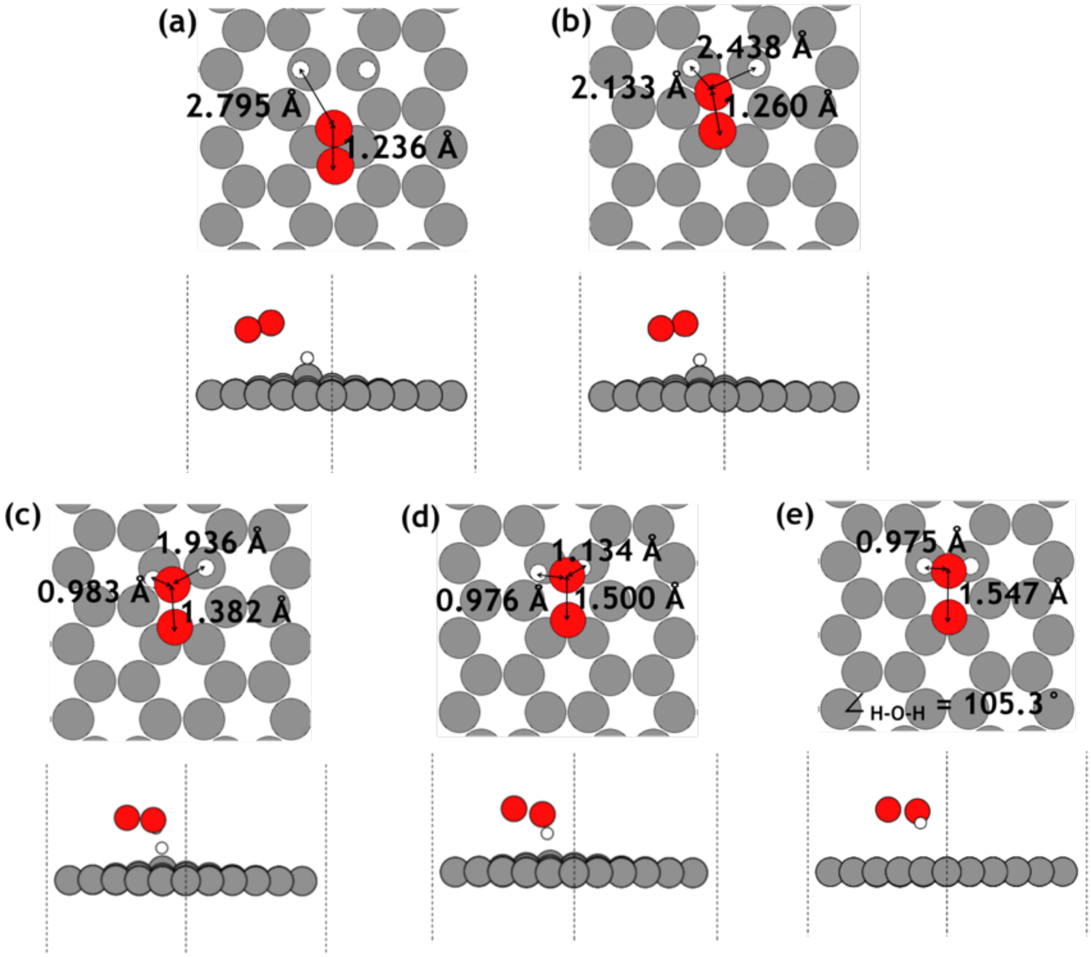

S14. Stability of various O* adsorption sites in vicinity of H dimer

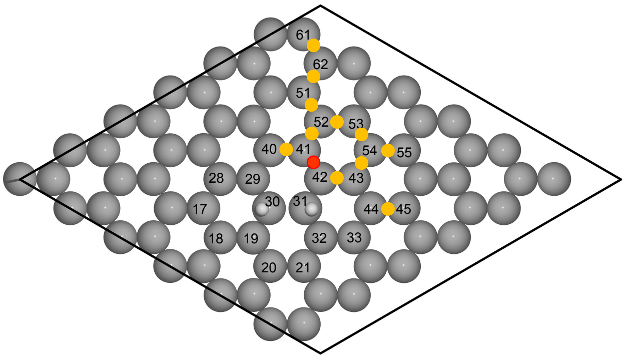

Site (bridge)	Δ*E* (eV)
41-42	0.00
44-45	0.42
42-43	0.45
40-41	0.71
51-62	0.71
51-52	0.83
53-54	0.85
41-52	0.86
50-51	0.87
43-54	0.88
61-62	0.90
54-55	0.92

Stability of various O* adsorption sites referenced to the most stable one (site 41-42 marked in red) in the vicinity of an H dimer. Meta-stable structures are marked in orange. For bonding of O* next to the graphene-like clusters we expect even higher stability due to the sp^3-like bonding geometry here.

T1. Calculated diffusion barriers for O and H atoms on graphene

Calculated diffusion barriers with various supercell sizes and k-point densities for O and H atoms adsorbed on freestanding graphene.

Diffusion barrier of single H* and O* (in eV)

Graphene size	K-points	ΔE(Hbri - Htop)	ΔE(O_{bri}- O_{top})
3x3	8x8	1.18	-0.82
4x4	6x6	1.14	-0.67
5x5	5x5	1.15	-0.66
6x6	4x4	1.06	-0.71
7x7	3x3	1.04	-0.62
8x8	3x3	1.09	-0.64
8x8	6x6	1.09	

REFERENCES

(1) Ng, M. L.; Balog, R.; Hornekær, L.; Preobrajenski, A. B.; Vinogradov, N. A.; Mårtensson, N.; Schulte, K. Controlling Hydrogenation of Graphene on Transition Metals. *J. Phys. Chem. C* **2010**, *114* (43), 18559–18565. https://doi.org/10.1021/jp106361y.

(2) Vinogradov, N. A.; Schulte, K.; Ng, M. L.; Mikkelsen, A.; Lundgren, E.; Mårtensson, N.; Preobrajenski, A. B. Impact of Atomic Oxygen on the Structure of Graphene Formed on Ir(111) and Pt(111). *J. Phys. Chem. C* **2011**, *115* (19), 9568–9577. https://doi.org/10.1021/jp111962k.
